# Destructive *Phytophthora* on orchids: current knowledge and future perspectives

**DOI:** 10.3389/fmicb.2023.1139811

**Published:** 2024-01-05

**Authors:** Tusar Kanti Bag, Pranab Dutta, Manjunath Hubballi, Ravpreet Kaur, Madhusmita Mahanta, Ardhendu Chakraborty, Gitasree Das, Madhusmita Kataky, Rajesh Waghunde

**Affiliations:** ^1^Division of Plant Pathology, Indian Agricultural Research Institute, New Delhi, India; ^2^CPGSAS, CAU (Imphal) Umiam, Imphal, India; ^3^Department of Plant Pathology, College of Horticulture Bagalkot, Bagalkot, Karnataka, India; ^4^Krishi Vigyan Kendra, Khowai, Tripura, India; ^5^Department of Plant Pathology, Assam Agricultural University, Jorhat, Assam, India; ^6^Krishi Vigyan Kendra-Kamrup, Azara, Assam Agricultural University, Guwahati, Assam, India; ^7^College of Agriculture, Navsari Agricultural University, Bharuch, Gujarat, India

**Keywords:** *Phytophthora*, Orchidaceae, destructive pathogen, epidemiology, integrated management

## Abstract

Anton de Bary first coined the genus, *Phytophthora*, which means “plant destroyer”, viewing its devastating nature on potatoes. Globally plants have faced enormous threat from *Phytophthora* since its occurrence. In fact, a century ago, *Phytophthorapalmivora* was first reported on Dendrobium maccarthiae in Sri Lanka. Since then, members of beautiful flowering crops of the family Orchidaceae facing the destructive threat of *Phytophthora*. Several *Phytophthora* species have been recorded to infect orchids with economic loss worldwide. To date, orchids are attacked by 12 species of *Phytophthora*. Five *Phytophthora* species (*P. palmivora, P. nicotianae, P. cactorum, P. multivesiculata, P. meadii*) are the major pathogenic Oomycetous Chromista” rather than true fungi frequently occurred on Orchidaceae. *Phytophthora palmivora* (having ~32 orchid host genera in 15 countries), *Phytophthora nicotianae* (having ~15 orchid host genera in 16 countries), *Phytophthora cactorum* (having ~43 orchid host genera in 6 countries), *Phytophthora multivesiculata* (having 2 orchid host genera in 5 countries) and *Phytophthora capsici* (having 2 orchid host genera in all Vanilla growing countries) are potential destroyers of Orchidaceae. Most of them are water loving Oomycetes cause disease in moist environments (> 80% RH) at 16–28°C. In artificially constructed orchidaria, anthropogenic factors are mostly contributed to the dissemination *Phytophthora* diseases in addition to many other factors. Water management, clean cultivation, and agro-chemicals are the major options for effective management of orchid *Phytophthora*, as the eco-friendly management options like development of resistant hybrids/cultivars, biological disease management, transgenic approaches, RNAi technology remained in the infant stage. In this review, we intended to highlight the insight of *Phytophthora* diseases associated with the orchid disease with reference to the historical aspect of the diseases, symptoms and signs, the pathogens, taxonomy, geographic distribution, host range within the Orchidaceae, pathogen identification, molecular diagnostics, mating types and races, management options and strategies and future perspectives.

## Graphical abstract

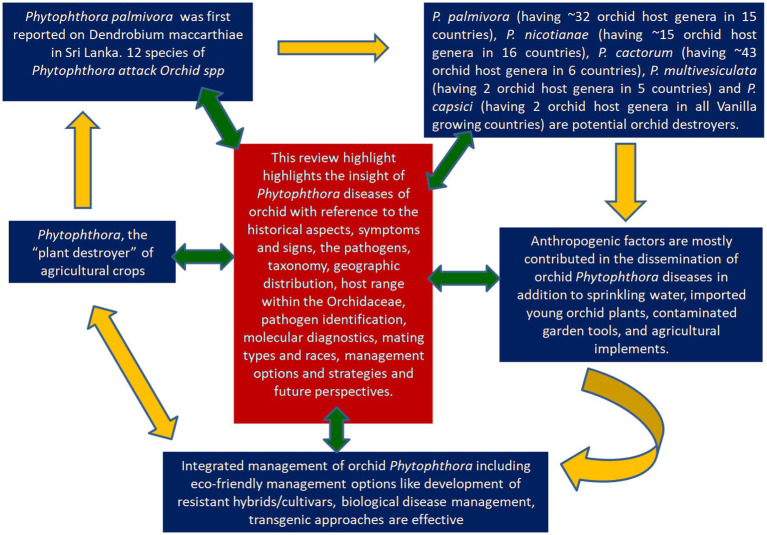

## Introduction

Orchids are economically highly valued ornamental flowering plants belonging to one of the largest plant families, Orchidaceae. These are traded commercially across the globe in the form of ornamental plants, cut flowers, and potted plants (Hinsley et al., 2018). As per the recent report, the total number of species is approximately 29,199 (Govaerts et al., 2017), and approximately 1, 25, 000 hybrid orchids are registered in the world (Jangyukala and Hemanta, 2021). They exhibit huge diversity with respect to the size, shape, and color of the flower. Furthermore, these are also popular for their durability and exquisite appearance. The value of the global orchid industry is approximately 400 billion US dollars (DITP News, 2015).

The advancement in commercial orchid production technology has increased the trade of orchid cut-flowers and potted plants several times around the world. However, the commercial production of orchids is challenged by an array of biotic as well as abiotic stresses across the globe. The biotic stresses comprise thrips, scale insects, mites, cockroaches, snails, slugs, nematodes, bacteria, fungi, and viruses, which infect the crop at different stages of production (Jensen, 1959; Farr et al., 1989; Hu et al., 1993; Uchida and Sipes, 1998). The two most important bacterial species infecting orchids are *Acidovorax avenae* subsp. *cattleyae* (Syn: *Pseudomonas cattleyae*), inciting bacterial brown spot disease (Miller, 1990), and *Pectobacterium chrysanthemi* (Syn: *Erwinia chrysanthemi*), inciting bacterial soft rot (Chan et al., 2005; Cating et al., 2008). Among the fungal diseases, black rot caused by species of *Phytophthora* and *Pythium,* anthracnose caused by species of *Colletotrichum*, *Fusarium* rots (Foster, 1955; Srivastava et al., 2018), flower spot (*Botrytis* sp.), and rusts are considered to be important. Major black rot caused by *Phytophthora* is known to be one of the most deadly diseases in orchids. The very name *Phytophthora* was described as a plant destructor or plant destroyer by de Bary (1876). There have been 116 species in total in the genus *Phytophthora*, infecting a range of crops across the globe. Several *Phytophthora* species have been recorded to inflict economic losses worldwide by the infection of orchids (Uchida, 1994; Erwin and Ribeiro, 1996; Orlikowski and Szkuta, 2006; Cating et al., 2009). Although the nature of the damage and economic losses depend on the growth stages and organs of infected orchid crops, global data on the intensity of the diseases in different countries and the actual losses incurred by different *Phytophthora* diseases on orchids have remained obscure. Furthermore, consolidated information on various aspects of the destructive *Phytophthora* diseases on orchids is lacking in the literature. This review aimed to highlight recent developments in *Phytophthora* diseases associated with orchids regarding symptoms and signs, the pathogens, taxonomy, geographic distribution, host range within the Orchidaceae, and management strategies, along with future perspectives.

## Historical perspective of *Phytophthora* species *infecting* Orchidaceae

There are several species of *Phytophthora* recorded among the various members of the family Orchidaceae. However, a brief historical aspect of important species of *Phytophthora* infecting orchids is enumerated below:

### *Phytophthora palmivora* Butler

**Figure 1:**
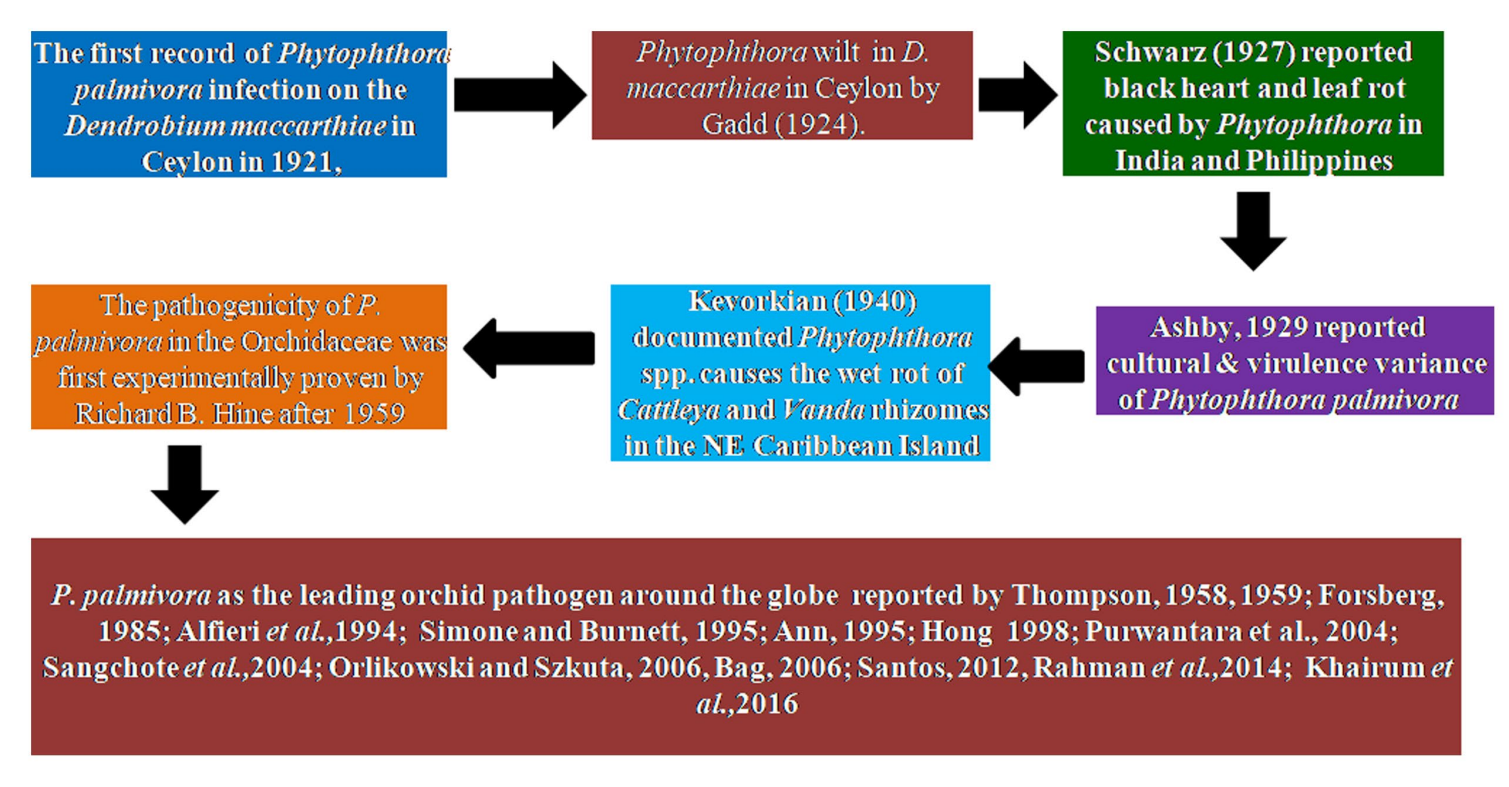
Historical perspective of *Phytophthora* species infecting orchidaceae.

The first record of *Phytophthora* infection on *Dendrobium maccarthiae* was reported from Ceylon (presently Sri Lanka) in 1921, in *ibid* which constitutes the oldest literature. The fungus was identified as *Phytophthora palmivora* (Petch, 1921) and reported to be responsible for the wilt disease of *D. maccarthiae* in Ceylon by Gadd (1924). Later, it was reported by Schwarz (1927) that several indigenous and imported orchid varieties from India and the Philippines were attacked by *Phytophthora* in Java (presently Indonesia). The disease was named black heart and leaf rot of orchids as characterized by the rotting of the heart and leaves with the final discoloration of affected areas and drooping of foliage (Figure 1; Supplementary File 1). Several species in Orchidaceae, *viz.*, *Cattleya* sp., *Dendrobium crumenatum*, *Grammatophyllum speciosum, Oncidium* sp., *Phalaenopsis amabilis, Phalaenopsis schilleriana*, *Vanda coerulea,* and *Vanda limbata,* were invaded by this fungus. The strains of the fungus varied significantly in cultural characteristics and virulence with reference to their hosts. Initially, this rot disease was believed to be caused by the tropical fungus *Phytophthora omnivora* de *Bary,* which was considered synonymous with *Phytophthora faberi,* but later on, the fungus was identified as *P. palmivora* by Ashby (1929a). Interestingly, a contemporary agronomist, Kopp (1930), described a disease of *Vanilla* (family: Orchidaceae) in Reunion that resulted in black lesions and rot on the stems, leaves, and pods. These spots contained abundant *Phytophthora* sporangia similar to those of *P. palmivora* Butler (=*P. faberi* Maubl. at that time). It might be the same lineage that caused the pod rot in *Cocoa*. Subsequently, Kevorkian (1940) documented that *Phytophthora* spp. caused the wet rot of *Cattleya* and *Vanda* rhizomes in the northeastern Caribbean island of Puerto Rico. However, the name “Black rot of orchids” was first coined by Rossetti (1943) while giving an account of a disease of *Cattleya* and *Vanda* in Puerto Rico and *Stanhopea saccata* in an unnamed locality. The name black rot is used to designate a disease caused by pythiaceous fungi of more than one species, mainly including the genera *Pythium* or *Phytophthora* (Limber, 1946). The symptoms of *P. palmivora* (Bult.) were adequately described on mature plants of *Vanda* orchids, and it was reported that *P. palmivora* was a parasite of orchids in Singapore (Thompson, 1959). The pathogenicity of *P. palmivora* in the Orchidaceae was first experimentally proven by Richard B. Hine, who was working as a plant pathologist at the University of Hawaii, USA. He demonstrated that plants in the orchid genera *Dendrobium, Cattleya, Epidendrum, Paphiopedilum,* and *Vanda* were susceptible to *P. palmivora* (Bult.) when inoculated with suspensions of zoospores or mycelium on wounded leaves. However, a natural infection of *Cattleya* and *Vanda* by *P. palmivora* in the field was reported in Hawaii (Hine, 1962). Thereafter, several scientific reports confirmed that *P. palmivora* (Bult.) is the leading orchid pathogen in various countries around the globe (Thompson, 1958, 1959; Forsberg, 1985; Alfieri et al., 1994; Ann, 1995; Simone and Burnett, 1995; Hong et al., 1998; Yeh et al., 1998; Portales, 2004; Purwantara et al., 2004; Sangchote et al., 2004; Bag, 2006; Orlikowski and Szkuta, 2006; dos Santos, 2012; Rahman et al., 2014; Khairum et al., 2016).

### *Phytophthora nicotianae* Breda de Haan

The black rot disease on the *Laelia* orchid was reported to be caused by *Phytophthora parasitica* Dastur (=*P. nicotianae* Breda de Haan) in Buenos Aires (Argentina) by Rossetti (1943) who first brought it to the attention of the scientific community. Vine blight and fruit rot of *Vanilla* (*V. fragrans*) caused by *Phytophthora parasitica* were further reported in Puerto Rico (Cibes and Childers, 1949). Subsequently, *Phytophthora parasitica* was repeatedly confirmed as a phytopathogen of orchids in predominant orchid-growing countries (Chen and Hsieh, 1978; Takahito et al., 1979; Forsberg, 1985; Wey, 1988; Uchida and Aragaki, 1991; Uchida, 1994; Ann, 1995; Zhang et al., 2006; Tao et al., 2011a; Daly et al., 2013).

### *Phytophthora cactorum* (Leb. and Cohn) Schröeter

The occurrence of *Phytophthora cactorum* on orchids was first reported by Burnett in Florida, USA, in a series of scientific reports (Burnett, 1957, 1958, 1965, 1974). The fungus *P. cactorum* was reported to cause seedling blight, black leaf, and heart rot in *Cattleya, Vanda, Epidendrum, Laelia, Oncidium, Vanilla,* and *Grammatophyllum* in Florida. Later, occurrences of *P. cactorum* on orchids were also reported in several other countries (Arnold, 1986; Uchida and Aragaki, 1991; Pereira et al., 1993; Uchida, 1994; Claudio, 2003).

### *Phytophthora multivesiculata* Ilieva, Man in ‘t Veld, Veenbaas-Rijks and R. Pieters

At the end of the 20th century, a new species of *Phytophthora* was isolated and characterized from blackened leaves and stems of naturally infected *Cymbidium* orchids in the Netherlands. The species was described as *Phytophthora multivesiculata* (Ilieva et al., 1998). Subsequently, the disease was reported exclusively on *Cymbidium* in New Zealand (Hill, 2004), Australia (Cunnington et al., 2009), Taiwan (Chern et al., 2011), and South Africa (Bose and Hammerbacher, 2022).

### Other species of *Phytophthora* reported on orchids

*Phytophthora erythroseptica* var. *erythroseptica* in Australia (Hall, 1989; Shivas, 1989), *Phytophthora cinnamomi* in Hawaii, USA (Uchida and Aragaki, 1991; Uchida, 1994), *Phytophthora meadii* in India (Bhai and Thomas, 2000; Bhai and Dhanesh, 2008*), Phytophthora capsici* in Indonesia (Andriyani et al., 2008), *Phytophthora citricola* in Taiwan (Ann, 2000), *Phytophthora syringae* in New South Wales, Australia (Cunnington et al., 2009), *Phytophthora tropicalis* in (Tahiti) French Polynesia (Aragaki and Uchida, 2001), *Phytophthora jatrophae* in French Polynesia and Puerto Rico (Bouriquet, 1954) as well as unidentified species of *Phytophthora* in Taiwan (Chern and Ann, 1996), Costa Rica (Rivera and Corrales, 2007), and Japan (Rahman et al., 2014) were found in the scientific literature. However, their frequency was found to be lower. Currently, it is globally accepted that five major *Phytophthora* species, *viz.*, *P. palmivora* Butler, *P. nicotianae* Breda de Haan, *P. cactorum* (Leb. and Cohn) Schröeter, *P. multivesiculata* Ilieva et al., and *P. meadii* are responsible for orchid diseases that are globally known by different vernacular names as black rot, crown rot, brown rot, stem and leaf rot, heart and leaf rot, top and shoot rot, and leaf spot, including seedling rot and damping off (Tao et al., 2011a). An elaborate list of species of *Phytophthora* infecting various members of Orchidaceae is presented in Table 1.

**Table 1:** List of *Phytophthora* species reported on Orchidaceae across the globe.

Sl. no.	Orchid host plant	*Phytophthora* species									
		*P. palmivora*	*P. nicotianae*	*P. cactorum*	*P. multivesiculata*	*P. erythroseptica*	*P. cinnamomi*	*P. megasperma*	*P. meadii*	*P. capsici*	*P. jatrophae*
1.	*Aerides* sp.	+	−	+	−	−	−	−	−	−	−
2.	*Arachnis* hybrid	+	−	−	−	−	−	−	−	−	−
3.	*Aranda* hybrid	+	−	−	−	−	−	−	−	−	−
4.	*Aranda* sp.	+	+	−	−	−	−	−	−	−	−
5.	*Aranthera* hybrid	+	−	−	−	−	−	−	−	−	−
6.	*Ascocenda* sp.	+	+	+	−	−	−	−	−	−	−
7.	*Brassavola* sp.	+	−	+	−	−	−	−	−	−	−
8.	*Brassocattleya* sp.	+	−	+	−	−	−	−	−	−	−
9.	*Brassolaeliocattleya* sp.	−	−	+	−	−	−	−	−	−	−
10.	*Broughtonia* sp.	+	−	−	−	−	−	−	−	−	−
11.	*Cattleya* hybrid	+	+	+	−	−	−	−	−	−	−
12.	*Cattleya* sp.	+	+	+	−	−	−	−	−	−	−
13.	*Cymbidium* sp.	+	+	+	+	+	+	+	−	−	−
14.	*Dendrobium* sp.	+	+	+	−	−	−	−	−	−	−
15.	*Dinema* sp.	−	−	−	−	−	−	−	−	−	−
16.	*Epidendrum* sp.	+	+	+	−	−	−	−	−	−	−
17.	*Galeandra* sp.	−	−	+	−	−	−	−	−	−	−
18.	*Gongora* sp.	+	−	+	−	−	−	−	−	−	−
19.	*Grammatophyllum* sp.	+			−	−	−	−	−	−	−
20.	*Laelia* sp.	+	+	+	−	−	−	−	−	−	−
21.	*Laeliocattleya* sp.	+	+	+	−	−	−	−	−	−	−
22.	*Masdevallia* sp.	−	−	+	−	−	−	−	−	−	−
23.	*Maxillaria* sp.	+	−	+	−	−	−	−	−	−	−
24.	*Miltonia* sp.	+	−	+	−	−	−	−	−	−	−
25.	*Oncidium* sp.	+		+	−	−	−	−	−	−	−
26.	*Paphiopedilum* sp.	+	+	+	−	−	−	−	−	−	−
27.	*Paphiopedilum* hybrid	+	−	−	−	−	−	−	−	−	−
28.	*Phaius* sp.	+	−	−	−	−	−	−	−	−	−
29.	*Phalaenopsis* sp.	+	+	−	−	−	−	−	−	−	−
30.	*Rhynchostylis* sp.	+	−	+	−	−	−	−	−	−	−
31.	*Ridleyara* sp.	+	−	−	−	−	−	−	−	−	−
32.	*Spiranthes* sp.	−	+	−	−	−	−	−	−	−	−
33.	*Schomburgkia* sp.	+	−	+	−	−	−	−	−	−	−
34.	*Sobralia* sp.	−	−	+	−	−	−	−	−	−	−
35.	*Sophronitis* sp.	+	−		−	−	−	−	−	−	−
36.	*Vanda* sp.	+	+	+	−	−	−	−	−	−	−
37.	*Vanilla* sp.	+	+	−	−	−	−	−	+	+	+
38.	*Zygopetalum* sp.	+	+	−	−	−	−	−	−	−	−
	Total	32	15	23	1	1	1	1	1	1	1

## Global distribution of *Phytophthora* species infecting orchids

**Figure 2:**
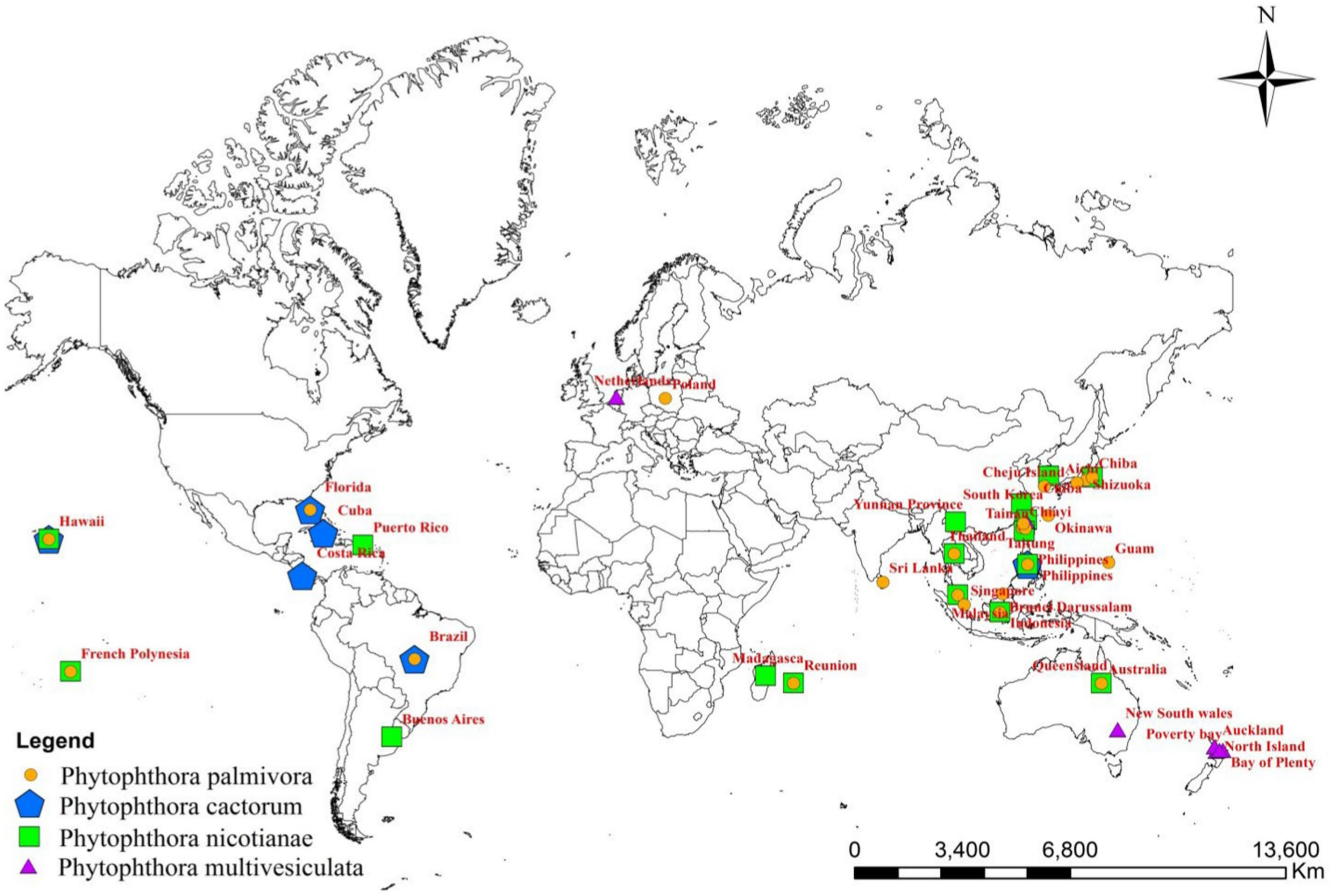
Global distribution of major *Phytophthora* species infecting orchids.

Although the genera and species of cultivated orchids are variable from continent to continent, orchids are globally distributed (Hinsley et al., 2018). When the orchid–fungi pathogen system is considered, *Phytophthora* cannot be separated from orchids. With the variation in the distribution of orchid hosts in various geographical locations, the distribution of *Phytophthora* spp. also varied widely on various continents (Figure 2).

Among the *Phytophthora* species documented as pathogens on orchids, *P. palmivora* appeared to be the most widely distributed one throughout the globe. *P. palmivora* has been recorded on one or more orchid hosts in multiple countries, such as Australia (Forsberg, 1985); Brazil (Santos dos Santos, 2012); Brunei Darussalam (Peregrine and Ahmad, 1982); Florida, USA (Alfieri et al., 1994; Simone and Burnett, 1995; Erwin and Ribeiro, 1996; Cating et al., 2008); French Polynesia (Mu and Tsao, 1987; Tsao and Mu, 1987); Guam, a territory of USA (Brown et al., 2007); Hawaii, USA (Hine, 1962; Uchida and Aragaki, 1991; Uchida, 1994); India (Erwin and Ribeiro, 1996); Java, Indonesia (Schwarz, 1927; Purwantara et al., 2004); Japan (Kiyomeda, 1992; Suzuki et al., 2008; Rahman et al., 2014); Korea Republic (Hong et al., 1998); Malaysia (Turner, 1966; Lim, 1980); Philippines (Ros, 1985; Divinagracia and Ros, 1986); Poland (Orlikowski and Szkuta, 2006; Orlikowski et al., 2008); Reunion (Kopp, 1930); Sri Lanka (Petch, 1921; Gadd, 1924; Zentmyer et al., 1973); Taiwan (Chen and Hsieh, 1978; Wey, 1988; Ann, 1995; Yeh et al., 1998; Hsu et al., 2002); and Thailand (Takahito et al., 1979; Sangchote et al., 2004; Khairum et al., 2016; Wongwan et al., 2021).

The distribution of *P. nicotianae* Breda de Haan is cosmopolitan (Cline et al., 2008). It has been reported on wide varieties of orchid species and hybrids in Argentina (Buenos Aires’ Rossetti, 1943); Australia (Forsberg, 1985; Daly et al., 2013); French Polynesia (Tsao and Mu, 1987; Hall, 1993); Hawaii, USA (Hine, 1962; Uchida and Aragaki, 1991; Uchida, 1994); Indonesia (Hall, 1993; Erwin and Ribeiro, 1996; Purwantara et al., 2004; Blair et al., 2008; Martin et al., 2014); Japan (Rahman et al., 2014); Korea (Cho and Shin, 2004); Madagascar (Erwin and Ribeiro, 1996); Malaysia (Liu, 1977); Mauritius (Wiehe, 1948; Orieux and Felix, 1968; Erwin and Ribeiro, 1996); the Philippines (Portales, 2004); Puerto Rico (Cibes and Childers, 1949; Erwin and Ribeiro, 1996); Reunion (Erwin and Ribeiro, 1996); Taiwan (Chen and Hsieh, 1978; Wey, 1988; Ann, 1995; Hsu et al., 2002); Thailand (Takahito et al., 1979); Yunnan Province, China (Tao et al., 2011a); Zhejiang province, China (Zhang et al., 2006; Li et al., 2008); and Hainan Island, China (Zeng et al., 2009).

Based on the pathogenic nature of Orchidaceae, the distribution of *P. cactorum* is restricted (Figure 1; Table 2) in Florida and New and Old-World countries (McMillan et al., 2009). Brazil (Pereira et al., 1993; Mendes et al., 1998); Costa Rica (Claudio, 2003); Cuba (Arnold, 1986); Florida, USA (Burnett, 1957; Burnett, 1965; Alfieri et al., 1994; Simone and Burnett, 1995; Cating et al., 2008; McMillan et al., 2010); and Hawaii, USA (Raabe et al., 1981; Uchida and Aragaki, 1991; Uchida, 1994) are major countries on the American continent where *P. cactorum* is frequently observed on orchids. Among Asian countries, *P. cactorum* has been occasionally recorded on orchids in India (Erwin and Ribeiro, 1996) and the Philippines (Schwarz, 1927; Erwin and Ribeiro, 1996).

**Table 2:** Host range of major *Phytophthora* species, including orchids.

*Phytophthora* species	Number of plant families infected	Number of plant genera infected	Number of genera infected within Orchidaceae
*Phytophthora palmivora*	60	160	32
*Phytophthora nicotianae*	90	225	15
*Phytophthora cactorum*	54	154	43
*Phytophthora multivesiculata*	1	2	2
*Phytophthora capsici*	28	51	2

As far as the orchid hosts are concerned, *P. multivesiculata* is mainly distributed in European countries, especially in the Netherlands (Ilieva et al., 1998; Figure 1; Table 2). Later, it was found in the North Island of New Zealand, prevalent in Northland, Auckland, the Bay of Plenty, and Poverty Bay, where Cymbidiums are grown commercially (Hill, 2004). Arguably, *P. multivesiculata* was also reported in Australia (Cunnington et al., 2009). In the recent past, an aberrant strain of *P. multivesiculata* was recorded in Taiwan (Chern et al., 2011). Very recently, the presence of *P. multivesiculata* was also documented in Pretoria, South Africa (Bose and Hammerbacher, 2022).

The presence of *P. erythroseptica* var. *erythroseptica* was recorded in Australia (Hall, 1989; Shivas, 1989), *P. cinnamomi* in Hawaii (Uchida and Aragaki, 1991; Uchida, 1994) and *Phytophthora megasperma var. megasperma* in New Zealand (Laundon, 1978; Boesewinkel, 1982).

*P. meadii* and *P. capsici* were established as pathogens of vanilla in India (Bhai and Thomas, 2000; Bhai and Dhanesh, 2008) and Indonesia (Andriyani et al., 2008), respectively; whereas *P. palmivora* was recorded in French Polynesia (Tsao and Mu, 1987) and Thailand (Sangchote et al., 2004) as *Vanilla* pathogen (Table 2). Unconfirmed reports of *Phytophthora tropicalis* in French Polynesia (Aragaki and Uchida, 2001) and *P. jatrophae* in French Polynesia and Puerto Rico (Bouriquet, 1954) as *Vanilla* pathogens were also available in the literature.

### Host range of *Phytophthora* species within the Orchidaceae

#### *Phytophthora palmivora* Butler

**Figure 3:**
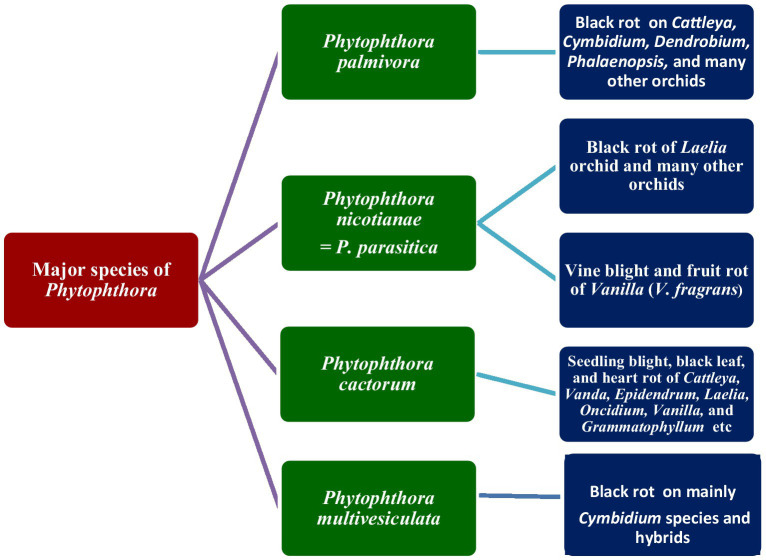
Major species of *Phytophthora* infecting orchids.

Many genera and species of orchids, along with their hybrids, are acknowledged as the hosts of *Phytophthora*. According to recent literature, *P. palmivora*, a prominent species infecting orchids, was reported to have a large number of hosts belonging to 160 genera of 60 plant families (Cline et al., 2008; Table 2; Figure 3; Supplementary File 2). Within the Orchidaceae, several species, hybrids, and intergeneric hybrids were reported to be infected by *P. palmivora* (Table 3). The major genera and species include *Aerides* sp. (Alfieri et al., 1994); *Arachnis* Maggie Oei (Lim, 1980); *Aranda* sp. (Turner, 1966); *Aranda* Christine, *Aranda* Deborah*, Aranda* Wendy Scott var. Greenfield (Lim, 1980), Aranda Wendy Scott (Tang, 1977); *Aranthera* James Storie (Lim, 1980); *Ascocenda* sp. (Alfieri et al., 1994); *Brassavola* sp. (Alfieri et al., 1994; Simone and Burnett, 1995); *Brassocattleya* sp. (Alfieri et al., 1994; Simone and Burnett, 1995; Cating et al., 2008); *Broughtonia* sp. (Alfieri et al., 1994; Simone and Burnett, 1995); *Cattleya* hybrids (Cating et al., 2008); *Cattleya skinneri* (Turner, 1966); *Cattleya* sp. (Schwarz, 1927; Hine, 1962; Turner, 1966; Zentmyer et al., 1973; Chen and Hsieh, 1978; Forsberg, 1985; Uchida and Aragaki, 1991; Alfieri et al., 1994; Ann, 1995; Simone and Burnett, 1995; Yeh et al., 1998; dos Santos, 2012; Rahman et al., 2014); *Cymbidium formosanum* (Ann, 1995); *Cymbidium hybridum* (Orlikowski and Szkuta, 2006); *Cymbidium owakensis* (Ann, 1995); *Cymbidium* sp. (Ann, 1995; Hong et al., 1998; Hsu et al., 2002; Orlikowski et al., 2008; Rahman et al., 2014); *Dendrobium bigibbum* (Rahman et al., 2014); *Dendrobium crumenatum* (Schwarz, 1927); *D. maccarthiae* (Petch, 1921; Gadd, 1924; Hine, 1962; Erwin and Ribeiro, 1996); *Dendrobium phalaenopsis* (Orlikowski and Szkuta, 2006); *Dendrobium* sp. (Peregrine and Ahmad, 1982; Forsberg, 1985; Uchida and Aragaki, 1991; Alfieri et al., 1994; Uchida, 1994; Ann, 1995; Orlikowski et al., 2008; Khairum et al., 2016); *Dinema polybulbon* (Suzuki et al., 2008; Rahman et al., _2014_); *Epidendrum* sp. (Hine, 1962; Uchida and Aragaki, 1991; Orlikowski and Szkuta, 2006; Orlikowski et al., 2008); *Gongora* sp. (Alfieri et al., 1994); *Grammatophyllum scriptum* (Schwarz, 1927); *Grammatophyllum speciosum* (Schwarz, 1927); *Laelia* sp. (Alfieri et al., 1994; Simone and Burnett, 1995); *Laeliocattleya* sp. (Turner, 1966; Uchida and Aragaki, 1991; Alfieri et al., 1994; Simone and Burnett, 1995; Cating et al., 2008); *Maxillaria* sp. (Alfieri et al., 1994); *Miltonia* sp. (Alfieri et al., 1994); *Oncidium* sp. (Schwarz, 1927; Kiyomeda, 1992; Alfieri et al., 1994; Ann, 1995; Rahman et al., 2014); *Paphiopedilum* sp. (Hine, 1962; Uchida and Aragaki, 1991; Alfieri et al., 1994; Ann, 1995); *Paphiopedilum* hybrid (Forsberg, 1985); *Phaius* sp. (Ann, 1995); *Phalaenopsis achilleriana, Phalaenopsis amabilis* (Schwarz, 1927); *Phalaenopsis aphrodite* (Anonymous, 1979); *Phalaenopsis lueddemanniana* (Orlikowski and Szkuta, 2006; Orlikowski et al., 2008); *Phalaenopsis* sp. (Ela, 1968; Wey, 1988; Alfieri et al., 1994; Ann, 1995); *Rhynchostyli*s *gigantea* (Wongwan et al., 2021); *Rhynchostyli*s sp. (Chen and Hsieh, 1978; Alfieri et al., 1994); *Ridleyara* sp. (Turner, 1966); *Schomburgkia* (Alfieri et al., 1994); *Sophronitis* sp. (Alfieri et al., 1994; Simone and Burnett, 1995); *Vanda coerulea* (Schwarz, 1927; Hine, 1962; Erwin and Ribeiro, 1996); *Vanda* hybrid cv. Bangkok Blue (Brown et al., 2007); *Vanda* hybrids (Lim, 1980); *Vanda lamellata* (Ros, 1985; Divinagracia and Ros, 1986); *Vanda limbata* (Schwarz, 1927); *Vanda* sp. (Thompson, 1958, 1959; Turner, 1966; Takahito et al., 1979; Peregrine and Ahmad, 1982; Forsberg, 1985; Uchida and Aragaki, 1991; Uchida, 1994; Ann, 1995; Purwantara et al., 2004; Rahman et al., 2014); *Vanilla planifolia* (Kopp, 1930; Mu and Tsao, 1987; Tsao and Mu, 1987; Sangchote et al., 2004); and *Zygopetalum mackayi* (Orlikowski and Szkuta, 2006).

**Table 3:** Host range of *Phytophthora palmivora* Butler, within Orchidaceae.

Host	Disease caused	Geographical distribution
*Aerides* sp.	black rot	Florida USA (Alfieri et al., 1994)
*Arachnis* Maggie Oei	root rot, crown rot	Malaysia (Lim, 1980)
*Aranda* Christine, *Aranda* Deborah*, Aranda* Wendy Scott var. Greenfield, *Aranda* Wendy Scott	root rot, crown rot, top rot (black rot)	Malaysia (Lim, 1980; Tang, 1977)
*Aranda* sp.	leaf blight	Malaysia (Turner, 1966)
*Aranthera* James Storie,	root rot, crown rot	Malaysia (Lim, 1980)
*Ascocenda* sp.	black rot,	Florida USA (Alfieri et al., 1994), Taiwan (Deng et al., 2015)
*Brassavola* sp.	black rot of leaf and heart rot	Florida USA (Alfieri et al., 1994; Simone and Burnett, 1995)
*Brassocattleya* sp.	black rot of leaf and heart rot	Florida USA (Alfieri et al., 1994; Simone and Burnett, 1995; Cating et al., 2008)
*Broughtonia* sp.	black rot of leaf and heart rot	Florida USA (Alfieri et al., 1994; Simone and Burnett, 1995)
*Cattleya* hybrids	black rot	Florida, USA (Cating et al., 2008)
*Cattleya skinneri*	leaf blight	Malaysia (Turner, 1966)
Cattleya sp.	leaf and heart rot, black rot, leaf blight, wilting, seedling death	Java (Schwarz, 1927); Hawaii, USA (Hine, 1962; Uchida and Aragaki, 1991), Florida, USA (Alfieri et al., 1994; Simone and Burnett, 1995); Sri Lanka (Zentmyer et al., 1973); Taiwan (Chen and Hsieh, 1978; Ann, 1995; Yeh et al., 1998); Australia (Forsberg, 1985); Brazil (South Bahia; dos Santos, 2012); Malaysia (Turner, 1966); Japan (Rahman et al., 2014)
*Cymbidium formosanum*	black rot, wilting, seedling death	Taiwan (Ann, 1995)
*Cymbidium hybridum*	blackening of leaves, stems, flowers, and root rots	Poland (Orlikowski and Szkuta, 2006)
*Cymbidium owakensis*	black rot, wilting, seedling death	Taiwan (Ann, 1995)
*Cymbidium* sp.	crown rot, leaf, and root rot	Taiwan (Ann, 1995; Hsu et al., 2002); Korea Republic (Hong et al., 1998); Poland (Orlikowski et al., 2008); Japan (Rahman et al., 2014)
*Dendrobium bigibbum*	black rot, leaf	Japan (Rahman et al., 2014)
*Dendrobium crumenatum*	leaf and heart rot	Java (Schwarz, 1927
*Dendrobium maccarthiae*	wilt, black rot, and leaf rot	Sri Lanka (Petch, 1921; Gadd, 1924); USA (Hine, 1962; Erwin and Ribeiro, 1996)
*Dendrobium phalaenopsis*	blackening of leaves, stems, flowers, and root rots	Poland (Orlikowski and Szkuta, 2006); Japan (Rahman et al., 2014)
*Dendrobium* sp.	black rot, leaf and root rot, seedling death, black necrosis on a cane, leaf blight, blossom spots	Florida USA (Alfieri et al., 1994); Australia (Forsberg, 1985), Thailand (Khairum et al., 2016); Poland (Orlikowski et al., 2008); Brunei Darussalam (Peregrine and Ahmad, 1982); Taiwan (Ann, 1995), Hawaii, USA (Uchida, 1994; Uchida and Aragaki, 1991)
*Dinema polybulbon*	black rot of leaf and pseudobulb	Japan (Suzuki et al., 2008; Rahman et al., 2014)
*Epidendrum* sp.	leaf and stem blight, leaf and root rot, seedling damping off, blackening of leaves, stems, and flowers	USA (Hine, 1962), Hawaii, USA (Uchida and Aragaki, 1991); Poland (Orlikowski and Szkuta, 2006; Orlikowski et al., 2008)
*Gongora* sp.	black rot	Florida, USA (Alfieri et al., 1994)
*Grammatophyllum scriptum*	leaf blight, heart rot	Java (Schwarz, 1927)
*Grammatophyllum speciosum*	leaf and heart rot	Java (Schwarz, 1927)
*Laelia* sp.	black rot of leaf and heart rot	Florida, USA (Alfieri et al., 1994; Simone and Burnett, 1995)
*Laeliocattleya* sp.	black foliar rots, root rot, heart rot, leaf blight seedling damping off	Malaysia (Turner, 1966); Hawaii, USA (Uchida and Aragaki, 1991), Florida USA (Alfieri et al., 1994; Simone and Burnett, 1995; Cating et al., 2008)
*Maxillaria* sp.	black rot	Florida, USA (Alfieri et al., 1994)
*Miltonia* sp.	black rot	Florida, USA (Alfieri et al., 1994)
*Oncidium* sp.	leaf and heart rot; black rot, wilting, seedling death; root and pseudobulb rot	Java (Schwarz, 1927); Taiwan (Ann, 1995), Japan (Kiyomeda, 1992; Rahman et al., 2014); Florida, USA (Alfieri et al., 1994)
*Paphiopedilum* sp.	leaf and stem blight; black foliar rots, root rot, seedling damping off; black rot, wilting, seedling death	Florida, USA (Alfieri et al., 1994); Taiwan (Ann, 1995); Hawaii, USA (Uchida and Aragaki, 1991); USA (Hine, 1962)
*Paphiopedilum* hybrid,	black foliar rots, root rot	Australia (Forsberg, 1985)
*Phaius* sp.	black rot, wilting, seedling death	Taiwan (Ann, 1995)
*Phalaenopsis schilleriana*	leaf and heart rot	Java (Schwarz, 1927)
*Phalaenopsis amabilis*	root rot, black rot, leaf, and heart rot	Indonesia (Schwarz, 1927), Java (Schwarz, 1927)
*Phalaenopsis aphrodite*	black rot and leaf rot	Taiwan (Anonymous, 1979)
*Phalaenopsis lueddemanniana*	blackening of leaves, stems, and flowers, leaf, and root rot	Poland (Orlikowski and Szkuta, 2006; Orlikowski et al., 2008)
*Phalaenopsis* sp.	black rot, wilting, seedling death	Philippines (Ela, 1968); Florida, USA (Alfieri et al., 1994); Taiwan (Wey, 1988; Ann, 1995)
*Rhynchostylis* sp.	black rot	Taiwan (Chen and Hsieh, 1978); Florida, USA (Alfieri et al., 1994)
*Rhynchostylis gigantea*	black rot	Thailand (Wongwan et al., 2021)
*Ridleyara* sp.	leaf blight	Malaysia (Turner, 1966)
*Schomburgkia* sp.	black rot,	Florida, USA (Alfieri et al., 1994);
*Sophronitis* sp.	black rot of leaf and heart rot	Florida, USA (Alfieri et al., 1994; Simone and Burnett, 1995)
*Vanda coerulea*	leaf rot	Java (Schwarz, 1927); USA (Hine, 1962), India (Erwin and Ribeiro, 1996)
*Vanda* hybrid cv. Bangkok Blue	black rot	Guam is a territory of the USA (Brown et al., 2007)
*Vanda* hybrids	root rot, crown rot	Malaysia (Lim, 1980)
*Vanda lamellata*	black rot	Philippines (Ros, 1985; Divinagracia and Ros, 1986)
*Vanda limbata*	leaf blight and heart rot	Java (Schwarz, 1927
*Vanda* sp.	black rot, wilting, seedling death, leaf blight, crown rot, blossom spots, and rots	Hawaii, USA (Uchida, 1994; Uchida and Aragaki,1991); Singapore (Thompson, 1958, 1959); Australia (Forsberg, 1985); Malaysia (Turner, 1966), Indonesia (Purwantara et al., 2004); Thailand (Takahito et al., 1979); Brunei Darussalam (Peregrine and Ahmad, 1982); Taiwan (Ann, 1995); Japan (Rahman et al., 2014)
*Vanilla planifolia*	black rot in roots, foot, stem, leaf, and pod	Reunion (Kopp, 1930); French Polynesia (Mu and Tsao, 1987; Tsao and Mu, 1987); Thailand (Sangchote et al., 2004)
*Zygopetalum mackayi*	blackening of leaves, stems, flowers, and root rots	Poland (Orlikowski and Szkuta, 2006)

#### *Phytophthora nicotianae* Breda de Haan

With its cosmopolitan distribution and polyphagous nature, *P. nicotianae* is known to have hosts in 255 genera in 90 plant families (Cline et al., 2008), including orchid hosts of at least 15 genera within the family Orchidaceae (Tables 1, 4). The orchid hosts reported to be infected by this pathogen are *Aranda* sp. (Liu, 1977); *Ascocenda* sp. (Takahito et al., 1979); *Cattleya* hybrid (Forsberg, 1985); *Cattleya* sp. (Hine, 1962; Uchida and Aragaki, 1991; Duff and Daly, 2002; Daly et al., 2013); *Cymbidium ensifolium* (Ann, 1995; Hsu et al., 2002); *Cymbidium kanran* (Cho and Shin, 2004); *Cymbidium rubrigemmum* (Ann, 1995; Hsu et al., 2002); *Cymbidium sinense* (Ann, 1995; Hsu et al., 2002); *Cymbidium* sp. (Uchida and Aragaki, 1991; Zeng et al., 2009); *Dendrobium aurantiaca* (Tao et al., 2011a); *Dendrobium candidum* (Zhang et al., 2006; Li et al., 2008); *Dendrobium chrysanthum* (Tao et al., 2011a); *Dendrobium chrysotoxum* (Tao et al., 2011a); *Dendrobium phalaenopsis* (Ann, 1995); *Dendrobium* sp. (Wiehe, 1948; Orieux and Felix, 1968; Forsberg, 1985; Uchida and Aragaki, 1991; Uchida, 1994; Ann, 1995; Erwin and Ribeiro, 1996); *Dendrobium thyrsiflorum* (Tao et al., 2011b); *Epidendrum* sp. (Uchida and Aragaki, 1991); *Grammatophyllum* sp. (Hall, 1993; Blair et al., 2008; Martin et al., 2014); *Grammatophyllum speciosum* (Erwin and Ribeiro, 1996); *Laelia* sp. (Rossetti, 1943); *Laeliocattleya* sp. (Uchida and Aragaki, 1991); *Paphiopedilum* sp. (Forsberg, 1985; Uchida and Aragaki, 1991); *Phalaenopsis* sp. (Chen and Hsieh, 1978; Wey, 1988; Ann, 1995); *Vanda* sp. (Hine, 1962; Takahito et al., 1979; Forsberg, 1985; Uchida and Aragaki, 1991; Duff and Daly, 2002; Daly et al., 2013; Rahman et al., 2014); *Vanilla fragrans* (Cibes and Childers, 1949); and *Vanilla planifolia* (Tsao and Mu, 1987; Hall, 1993; Erwin and Ribeiro, 1996; Portales, 2004; Purwantara et al., 2004; Zeng et al., 2009).

**Table 4:** Host range of *Phytophthora nicotianae* Breda de Haan within Orchidaceae.

Host	Disease caused	Geographical distribution
*Aranda* sp.	black rot	Malaysia (Liu, 1977)
*Ascocenda* sp.	black rot	Thailand (Takahito et al., 1979)
*Cattleya* hybrid	shoot rot	Australia (Forsberg, 1985)
*Cattleya* sp.	seedling blight, leaf and flower spots, leaf, heart, and stem rot and black rot, and pseudobulb/ shoot rot	Hawaii, USA (Hine, 1962); Australia (Duff and Daly, 2002, Daly et al., 2013); Hawaii, USA (Uchida and Aragaki, 1991); Taiwan (Deng et al., 2015)
*Cymbidium ensifolium*	leaf blight/black rot/death of the plant	Taiwan (Ann, 1995; Hsu et al., 2002)
*Cymbidium kanran*	black rot	Korea (Cho and Shin, 2004)
*Cymbidium rubrigemmum*	leaf blight/black rot/death of the plant	Taiwan (Ann, 1995; Hsu et al., 2002)
*Cymbidium sinense*	leaf blight/black rot/death of the plant	Taiwan (Ann, 1995; Hsu et al., 2002)
*Cymbidium* sp.	root rot, leaf blight	Hawaii, USA (Uchida and Aragaki, 1991; Zeng et al., 2009)
*Dendrobium aurantiacum*	root and basal stem rot leading to blight	Yunnan Province, China, (Tao et al., 2011b)
*Dendrobium candidum*	root rot, wilting, leading to blight	Zhejiang province, China (Zhang et al., 2006; Li et al., 2008)
*Dendrobium chrysanthum*	root and basal stem rot leading to blight	Yunnan Province, China (Tao et al., 2011b)
*Dendrobium chrysotoxum*	root and basal stem rot leading to blight	Yunnan Province, China (Tao et al., 2011b)
*Dendrobium thyrsiflorum*	root and basal stem rot leading to blight	Yunnan Province, China (Tao et al., 2011b)
*Dendrobium phalaenopsis*	leaf blight/black rot/death of the plant	Taiwan (Ann, 1995)
*Dendrobium* sp.	top rot, stem/shoot rot, seedling blight, leaf and flower spots, leaf, heart, and stem rot, and black rot	Australia (Forsberg, 1985); Mauritius (Wiehe, 1948; Orieux and Felix, 1968; Erwin and Ribeiro, 1996); Hawaii, USA (Uchida and Aragaki, 1991; Uchida, 1994); Taiwan (Ann, 1995)
*Epidendrum* sp.	leaf, stem rot, and black rot	Hawaii, USA (Uchida and Aragaki, 1991)
Grammatophyllum sp.	black rot	Indonesia (Hall, 1993; Blair et al., 2008; Martin et al., 2014).
Grammatophyllum speciosum	black rot	Indonesia (Erwin and Ribeiro, 1996)
*Laelia* sp.	black rot	Argentina (Buenos Aires; Rossetti, 1943)
*Laeliocattleya* sp.	leaf, heart, and stem rot and black rot	Hawaii, USA (Uchida and Aragaki, 1991)
*Oncidium flexuosum*	Leaf and pseudo-stem rot	Taiwan (Deng et al., 2015)
*Paphiopedilum* sp.	stem rot and black rot	Hawaii, USA (Uchida and Aragaki, 1991)
*Paphiopedilum* sp.	top rot, stem/shoot rot	Australia (Forsberg, 1985), Taiwan (Deng et al., 2015)
*Phalaenopsis* sp.	leaf blight/black rot/death of the plant	Taiwan (Wey, 1988; Chen and Hsieh, 1978; Ann, 1995)
*Spiranthes sinensis*	Leaf and pseudobulb rot	Taiwan (Deng et al., 2015)
*Vanda* sp.	top rot, stem rot seedling blight, leaf and flower pots, leaf, heart, and stem rot and black rot, *Phytophthora* rot	Japan (Rahman et al., 2014); Hawaii, USA (Hine, 1962); Hawaii, USA (Uchida and Aragaki, 1991); Thailand (Takahito et al., 1979); Australia (Forsberg, 1985; Duff and Daly, 2002; Daly et al., 2013); Japan (Rahman et al., 2014)
*Vanilla fragrans*	vines blight and fruit rot	Puerto Rico (Cibes and Childers, 1949)
*Vanilla planifolia*	root rot, vine rot, leaf and bean blacking, pod rot	Indonesia (Purwantara, et al., 2004; Erwin and Ribeiro, 1996); Philippines (Drenth and Guest, 2004); Madagascar (Erwin and Ribeiro, 1996); Reunion (Erwin and Ribeiro, 1996); French Polynesia (Tsao and Mu, 1987; Hall, 1993); Puerto Rico (Erwin and Ribeiro, 1996); China (Zeng et al., 2009)

#### *Phytophthora cactorum* (Leb. and Cohn) Schröeter

*Phytophthora cactorum* (Leb. and Cohn) Schröeter is another phytopathogen of crop plants, consisting of 154 genera in 54 plant families (Cline et al., 2008), including 22 genera of orchids within the family Orchidaceae (Tables 1, 5). Reported orchid hosts are *Aerides* sp. (Alfieri et al., 1994); *Ascocenda* sp. (Alfieri et al., 1994); *Brassavola* sp. (Alfieri et al., 1994; Simone and Burnett, 1995); *Brassocattleya* sp. (Cating et al., 2008); *Brassolaeliocattleya* sp. (Alfieri et al., 1984); *Cattleya aurantiaca* (Claudio, 2003); *Cattleya dowiana* (Claudio, 2003); *Cattleya* hybrids (Claudio, 2003; Cating et al., 2008); *Cattleya* sp. (Schwarz, 1927; Burnett, 1957, 1965; Arnold, 1986; Pereira et al., 1993; Simone and Burnett, 1995; Mendes et al., 1998; McMillan et al., 2010);*Cattleya skinneri* (Claudio, 2003); *Cymbidium* sp. (Uchida and Aragaki, 1991; Uchida, 1994); *Dendrobium* sp. (Alfieri et al., 1994); *Epidendrum* sp. (Alfieri et al., 1984); *Galeandra baueri* (Miller, 1990); *Gongora* sp. (Alfieri et al., 1994); *Grammatophyllum* sp.(Erwin and Ribeiro, 1996); *Laelia* sp. (Simone and Burnett, 1995); *Laeliocattleya* sp. (Raabe et al., 1981; Simone and Burnett, 1995; Cating et al., 2008);*Masdevallia* sp. (Claudio, 2003); *Maxillaria* sp. (Alfieri et al., 1994); *Miltonia* sp. (Alfieri et al., 1994); *Oncidium* sp. (Alfieri et al., 1994); *Paphiopedilum* sp. (Alfieri et al., 1994); *Rhynchostylis* sp. (Alfieri et al., 1994); *Schomburgkia* sp. (Alfieri et al., 1994; Simone and Burnett, 1995); *Schomburgkia undulata* (Claudio, 2003); *Sobralia macrantha* (Claudio, 2003); *Vanda* sp. (Alfieri et al., 1984; Erwin and Ribeiro, 1996); and *Vanda coerulea* (Erwin and Ribeiro, 1996).

**Table 5:** Host range of *Phytophthora cactorum* (Leb. and Cohn) Schröeter within Orchidaceae.

Host	Disease caused	Geographical distribution
*Aerides*	Black rot	Florida USA (Alfieri et al., 1994)
*Ascocenda*	Black rot	Florida USA (Alfieri et al., 1994)
*Brassavola*	Black rot	Florida USA (Alfieri et al., 1994; Simone and Burnett, 1995
*Brassocattleya*	Black rot of leaf and heart rot	Florida USA (Cating et al., 2008)
*Brassolaeliocattleya* sp.	Root rot and black rot	Florida, USA (Alfieri et al., 1984)
*Cattleya aurantiaca*	Roots and rhizome rot	Costa Rica (Claudio, 2003)
*Cattleya dowiana*	Roots and rhizome rot	Costa Rica (Claudio, 2003)
*Cattleya hybrids*	Black rot, roots and pseudobulb rot, leaf blight	Florida USA (Cating et al., 2008), Costa Rica (Claudio, 2003)
*Cattleya* sp.	Burning or black rot of orchids, Leaf blight	Philippines (Schwarz, 1927); Brazil (Pereira et al., 1993; Mendes et al., 1998); Cuba (Arnold,1986); Florida USA (Burnett, 1957; Burnett, 1965; Simone and Burnett, 1995; McMillan et al., 2010)
*Cattleya skinneri*	Roots and rhizome rot	Costa Rica (Claudio, 2003)
*Cymbidium* sp.	Black rot, leaf blight	Hawaii, USA (Uchida and Aragaki, 1991; Uchida, 1994)
*Dendrobium* sp.	Black rot	Florida USA (Alfieri et al., 1994)
*Epidendrum* sp.	Black rot, leaf blight	Florida, USA (Alfieri et al., 1984)
*Galeandra baueri*	Root rot and black rot	Florida, USA (Miller, 1990)
*Gongora*	Black rot	Florida USA (Alfieri et al., 1994)
*Grammatophyllum* sp.	Stem rot, Leaf blight	Philippines (Erwin and Ribeiro, 1996)
*Laelia*	Black rot, pseudobulb rot	Florida USA (Simone, and Burnett, 1995)
*Laeliocattleya* sp.	Root rot, black rot of leaf and heart rot	Hawaii, USA (Raabe et al., 1981); Florida USA (Simone and Burnett, 1995; Cating et al., 2008)
*Masdevallia* sp.	Roots and rhizome rot	Costa Rica (Claudio, 2003)
*Maxillaria*	Black rot	Florida USA (Alfieri et al., 1994)
*Miltonia*	Black rot	Florida USA (Alfieri et al., 1994)
*Oncidium*	Black rot	Florida USA (Alfieri et al., 1994)
*Paphiopedilum* sp.	Black rot	Florida USA (Alfieri et al., 1994)
*Rhynchostylis* sp.	Black rot	Florida USA (Alfieri et al., 1994)
*Schomburgkia*	Black rot	Florida, USA (Alfieri et al., 1994; Simone and Burnett, 1995);
*Schomburgkia undulata*	Roots and rhizome rot	Costa Rica (Claudio, 2003)
*Sobralia macrantha*	Roots and rhizome rot	Costa Rica (Claudio, 2003)
*Vanda* sp.	Stem and leaf rot; pseudobulb rot, leaf blight	Florida, USA (Alfieri et al., 1984); Philippines (Erwin and Ribeiro, 1996)
*Vanda coerulea*	Black rot, stem, and leaf blight	India (Erwin and Ribeiro, 1996)

#### *Phytophthora multivesiculata* Ilieva et al.

*Phytophthora multivesiculata* was first reported on the boat orchid *Cymbidium* in the Netherlands (Ilieva et al., 1998). This fungal species is known to have a restricted host only in Orchidaceae to date (Table 6). Therefore, it exhibits pathogenicity only against species of *Cymbidium*. The reported hosts are *Cymbidium* sp. (Ilieva et al., 1998; Hill, 2004; Cunnington et al., 2009; Bose and Hammerbacher, 2022), namely *Cymbidium tracyanum* (Chern and Ann, 1996); *Cymbidium hybridum, Cymbidium ensifolium, Cymbidium sinense,* and *Cymbidium rubrigemmum* (Chern et al., 2011).Very recently, *P. multivesiculata* was documented to cause black rot on the Leopard orchid *Ansellia africana* in Pretoria, South Africa (Bose and Hammerbacher, 2022).

**Table 6:** Host range of *Phytophthora multivesiculata* Ilieva et al. within Orchidaceae.

Host	Disease caused	Geographical distribution
*Cymbidium* sp.	Dry leaf rot, leaf blotch, and rot, blackening of leaf and stem	Netherlands (Ilieva et al., 1998); New Zealand (Hill, 2004); Australia (Cunnington et al., 2009); South Africa (Bose and Hammerbacher, 2022)
*Cymbidium tracyanum*	Black rot and drying	Taiwan (Chern and Ann, 1996
*Cymbidium hybridum*	Black rot	Taiwan (Chern et al., 2011)
*Cymbidium ensifolium*	Black rot	
*Cymbidium sinense*	Black rot	
*Cymbidium rubrigemmum*	Black rot	
*Ansellia africana*	Black rot	South Africa (Bose and Hammerbacher, 2022)

## Diversity of symptoms and signs on orchids by different species of *Phytophthora*

Since the first report of *Phytophthora palmivora* occurrence on *D. maccarthiae* in Ceylon (Petch, 1921) causing wilt (Gadd, 1924) and an adequate description of symptoms of the disease caused by this pathogen on seedlings or matured hybrids of *Vanda* orchids in Singapore, numerous reports of *Phytophthora* diseases on orchids have been accumulated (Thompson, 1959). Summing up all the *up-to-date* literature on *Phytophthora* diseases of orchids, it is evident that *Phytophthora* generates a variety of symptoms in infected plants. According to the observation of Orlikowski and Szkuta (2006), symptoms may vary with the age of host plants, species, and organs attacked. In reality, major *Phytophthora* diseases of orchids are widely known as black rot (Ann, 1995; Erwin and Ribeiro, 1996; Yeh et al., 1998; Brown et al., 2007; Cating et al., 2010; Chern et al., 2011), wilt (Gadd, 1924; Hine, 1962), crown rot (Hong et al., 1998), heart and leaf rot (Schwarz, 1927; Hine, 1962; Chen and Hsieh, 1978), stem and leaf rot (Orlikowski and Szkuta, 2006), top and shoot rot (Daly et al., 2013), roots and rhizome rot (Claudio, 2003), blight (Li et al., 2008), bud and flower blight (Uchida, 1994), and seedling blight (Tao et al., 2011a) or damping off (Uchida and Aragaki, 1991; Uchida, 1994) in different countries. All these orchid diseases caused by *Phytophthora* spp. are known by the common name *Phytophthora* rots. Variation in the name of the disease is based on the severity of the disease symptoms on specific organs of the orchid hosts. The symptoms of major species are presented here.

### Phytophthora palmivora

Uchida has a long history of involvement with the research of orchid diseases at the University of Hawaii in Honolulu, USA, and has successfully enumerated various symptoms of *Phytophthora* diseases on a galaxy of tropical orchids, covering several phenological stages of crop growth from seedling to budding and flowering (Uchida, 1994). Symptoms of leaf and pseudobulb rot caused by *P. palmivora* on *Cattleya, Laeliocattleya,* and related hybrids are often dark brown to black. After the initiation of leaf infection, the fungus moves quickly through the leaves and pseudobulb; the leaves turn black within a few weeks and fall. Before the fall of the leaf, infection is initiated in the pseudobulbs, which causes gradual rot of the pseudobulbs where the fungus harbors for longer periods. Young plants are killed rapidly, but adult plants decline gradually. In community pots, seedling blight or damping is also observed due to overcrowding (Uchida and Aragaki, 1991).

**Figure 4:**
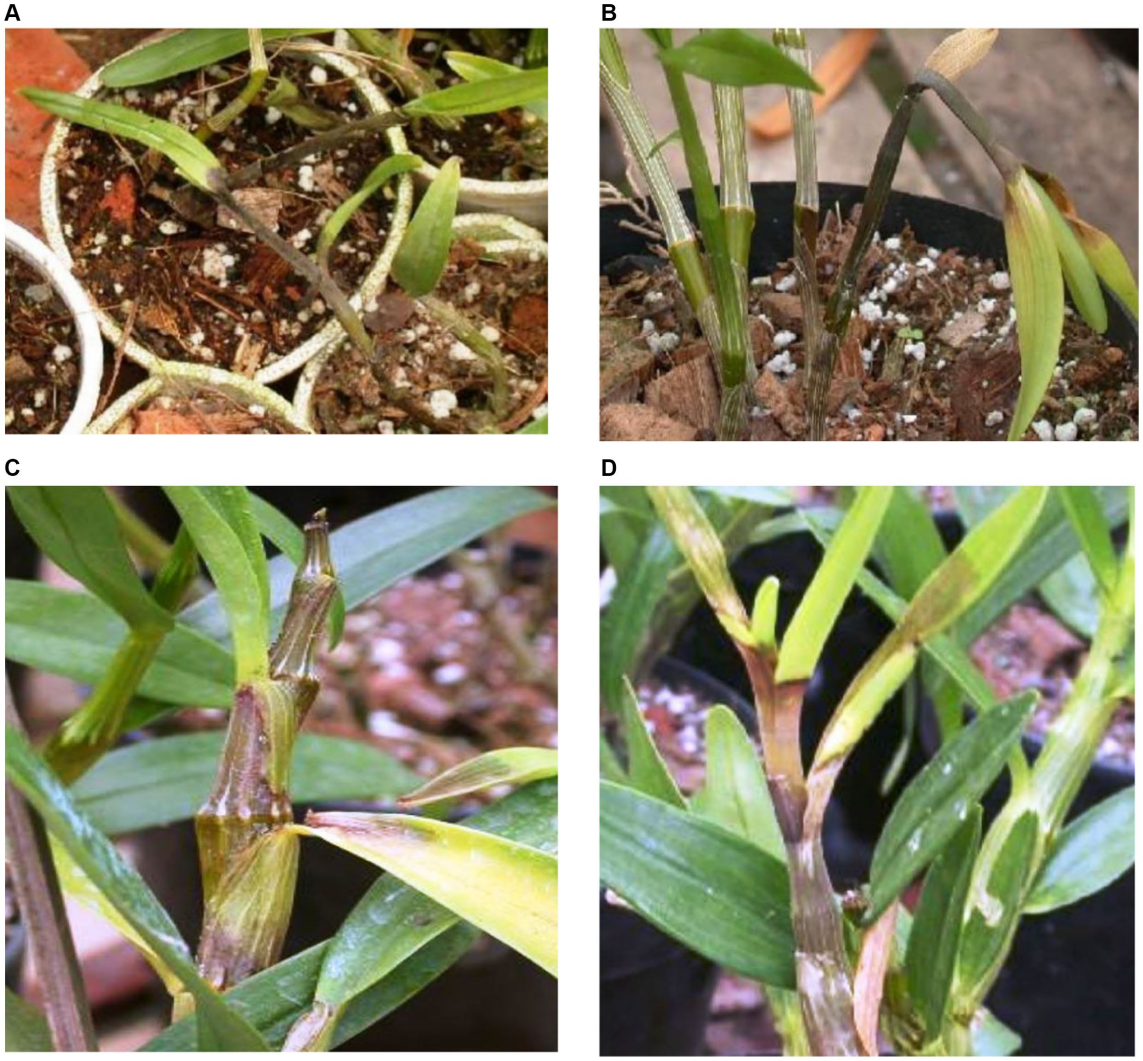
*Phytophthora* rot on *Dendrobium nobile* in different growth stages. **(A)** Damping off symptoms in community pot during hardening of tissue cultured plantlets. **(B)** Rotting of side shoots in the potted plan. **(C)** Matured plant showing progressing rotting of stems and defoliation. **(D)** Matured plant showing rotting on top of the growing plants.

**Figure 5:**
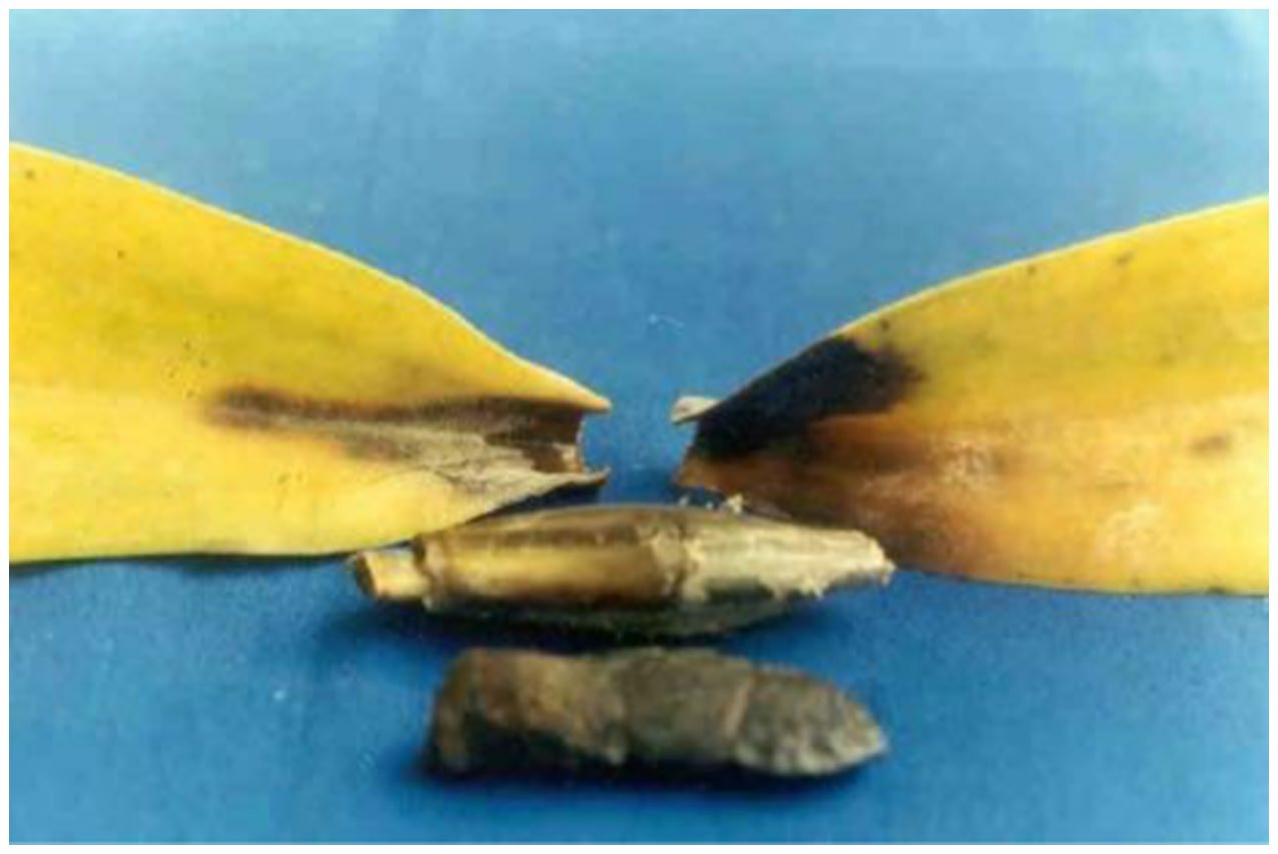
Symptom of black rot caused by *Phytophthora* on *Cattleya* hybrid.

**Figure 6:**
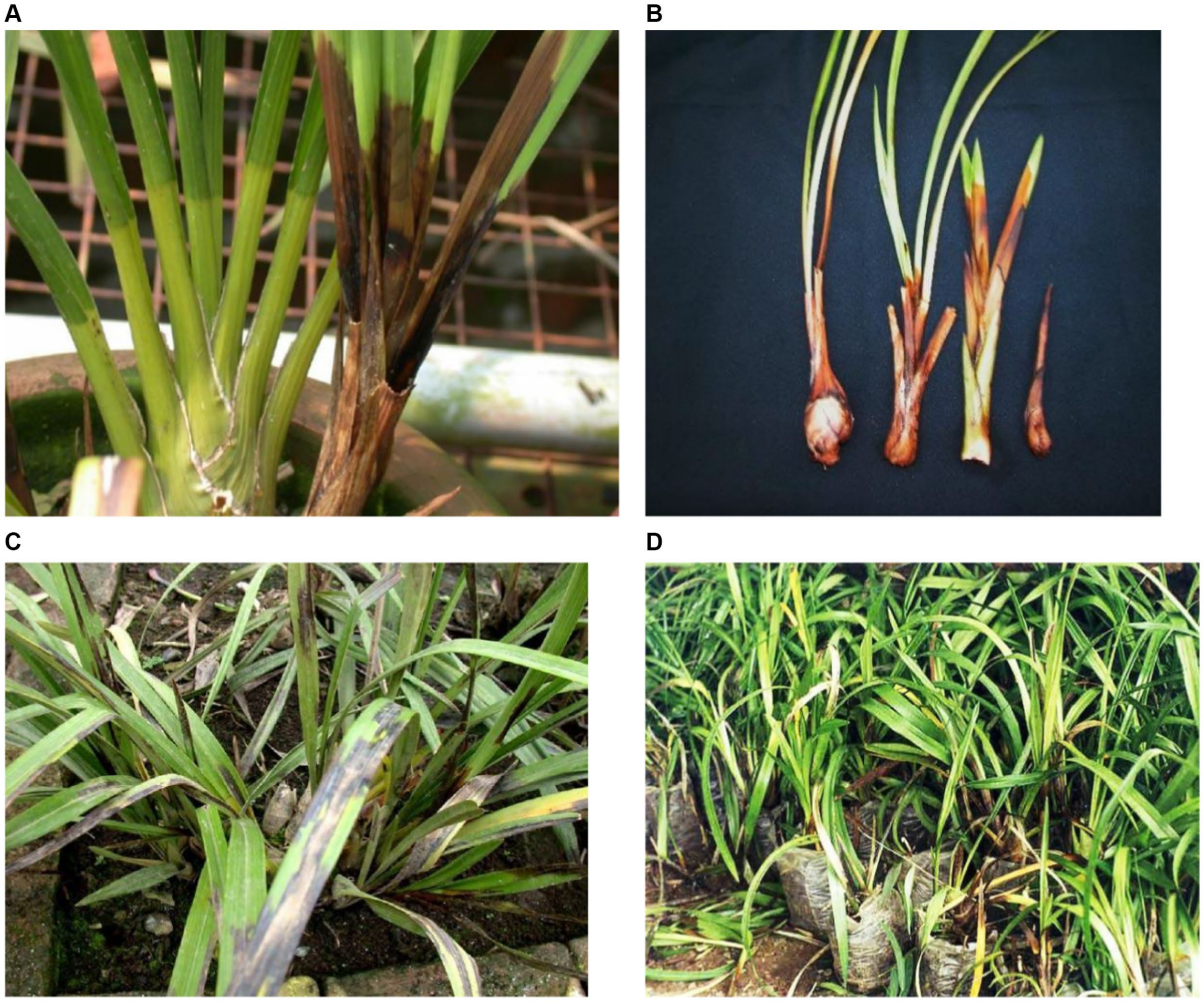
**(A)** New shoot rot caused by *Phytophthora* in potted *Cymbidium* orchid. **(B)** *Phytophthora* infected shoots of different ages turning black. **(C)** Black leaf blight incited by *Phytophthora* on *Cymbidium* leaves. **(D)** Mass destruction of *Phytophthora*-infected *Cymbidium* orchids in Nursery.

Blossom, bud, and spike rots were also caused by *P. palmivora* in *Dendrobium* and *Vanda* orchids. It results in the production of light brown watery lesions with slightly darker centers on flowers (Thompson, 1958, 1959; Uchida and Aragaki, 1991) which may be similar to that of *Botrytis* flower spots (gray mold). However, gray mold with a mass of powdery gray spores is not present in the case of *Phytophthora* blight or flower spots (Uchida and Aragaki, 1991). The sign of *Phytophthora* crown rot in the sympodial orchid, *Cymbidium*, starts at the basal region of an infected plant and progresses upward to the lower leaves. Subsequently, typical water-soaked lesions appeared on the lower leaves, and the plant became wilted, blighted, and died. A study by Orlikowski and Szkuta (2006) recorded symptoms of *P. palmivora* rot in Polish greenhouse orchids based on their observation of *Cymbidium, Dendrobium phalaenopsis, Epidendrum*, *Phalaenopsis lueddemanniana,* and *Zygopetalum mackayi*. *P. palmivora* mostly attacks the leaves that gradually spread onto the stems and roots, and its symptoms are evident as irregular yellow-brown or black areas in different parts of the plants. On *Phalaenopsis,* yellow to brown tongue-shaped spots appeared mainly on the leaf base, while they spread upward, invading the subsequent upper leaves that rot and die. Furthermore, the infection spreads to the next higher leaf, resulting in the detachment of leaves from the main plants after turning brown to black. When the disease moves downward, it ultimately reaches the roots, causing root rots. As per our knowledge, no report of rots on the flower was reported from Poland. Rotting was observed in tissue culture seedlings of *Dendrobium nobile* in community pots during hardening. Furthermore, rotting in mature potted plants started from the base with gradual progression to the top, causing defoliation (Figures 4A–D). Severely infected *Cattleya* plants turn black; leaves and stems get detached from each other (Figure 5). On *Cymbidium,* severe black rot symptoms on new side shoots, leaves, and pseudobulbs and mass destruction of orchid plants were reported (Figures 6A–D) in Darjeeling and Sikkim, India (Bag, 2006).

### Phytophthora nicotianae

The earliest report of black rot disease on *Laelia* orchids was published by Rossetti (1943). The study described distinct symptoms of the disease caused by *Phytophthora parasitica* (= *nicotianae*) in Buenos Aires (the capital city of Argentina). The disease was characterized by a dark brown, flaccid rot of the pseudobulb tissues and sharply defined discoloration of the leaves, accompanied by sharply defined discoloration of the leaves. The infected leaves get detached from the main plant and the fungus further progresses until they are completely blackened. The pseudobulbs initially remain attached to the rhizome; however, later on, they wither as the infection progresses. As observed by Takahito et al. (1979), the infected parts such as buds, leaves, and stems of *Vanda* and *Ascocenda* with *P. nicotianae* show light brown to brown water-soaked lesions that turn into dark brown to black, followed by the death of the plants eventually. Subsequently, similar symptoms were also reported in various orchid hosts from many other countries (Uchida and Aragaki, 1991; Uchida, 1994; Ann, 1995; Duff and Daly, 2002; Daly et al., 2013). The seedling blight of *Dendrobium candidum* caused by *P. nicotianae* was reported from Zhejiang province, China (Zhang et al., 2006; Li et al., 2008). Typical root and basal stem rot symptoms that progress in an acropetal direction, causing the death of plants in a few days, were recorded in hardened tissue cultured seedlings of *Dendrobium* in the field. The rotten tap roots show brownish-black to black sunken lesions that frequently extend up to the stems. Typical brownish-black lesions are visible in older plants on the tender top parts, causing top wilt and defoliation. Later, Tao et al. (2011a) reported similar root and basal stem rot leading to blight and defoliation caused by *P. nicotianae* on *D. aurantiacum, D. chrysanthum, D. thyrsiflorum,* and *D. chrysotoxum* in Yunnan Province, China.

### Phytophthora cactorum

In Florida, USA, *P. cactorum* has been reported to cause seedling blight, black leaf, and heart rot in *Cattleya, Vanda, Epidendrum, Laelia, Oncidium, Vanilla*, and *Grammatophyllum* (Burnett, 1957, 1958, 1965, 1974). Unlike Hawaii, *P. cactorum* is a major pathogen of *Cattleya* species and its hybrids in Florida and New and Old-World countries. This fungus infects leaves, pseudobulbs, rhizomes, and flower buds (McMillan et al., 2010). The symptomatic expression of black rot disease caused by *P. cactorum* on the *Cattleya* species (*C. skinneri* and *C. aurantiaca*), including its hybrids, was enumerated by Claudio (2003) in the Central Valley of Costa Rica. The disease was also found to attack *Schomburgkia undulata, Masdevallia,* and *Sobralia macrantha*.

The infection initiates in the roots and rhizomes; later, the fungus spreads to the aerial parts of all the infected plants, causing decay and dark discoloration. The characteristic symptoms of the disease include immediate detachment of the leaves from the main stem or the leaves becoming brittle at the point of attachment to pseudobulbs and ultimately falling on the ground or the growing medium. The leaves become necrotic and develop a thin, floury layer on the plant surface, which has sporangia of the fungus under highly moist conditions.

### Phytophthora multivesiculata

As per current knowledge, *P. multivesiculata* is reported to be the pathogen of only *Cymbidium* orchids, and no other host. During the formation of the new species “*multivesiculata*” under the genus “*Phytophthora,”* the original symptom of rot disease caused by *P. multivesiculata* on naturally infected *Cymbidium* in the Netherlands was described by Ilieva et al. (1998). Naturally infected plants were recorded to have dry rot of leaves that showed a change in color to brown with typical horizontal zebra CV-like stripes (approximately 0.5 cm wide). A lighter discoloration in the middle and a dark brown to black color in the margin was observed. The base of the pseudobulbs showed water-soaked brownish-black discolored tissue. Under artificial inoculation, a typical brown rot was visible in 6 days, but the typical zebra-like stripes were not as prominent as a natural infection on *Cymbidium* leaves. The symptoms were found to be more or less similar on mature *Cymbidium* leaves and pseudobulbs in New Zealand. A characteristic internal, blue-black, or purplish-brown discoloration along with a sour odor was observed in infected young pseudobulbs. The roots remained grayish-white, unaffected while the rest of the plant had turned brown (Hill, 2004). In Taiwan, Chern et al. (2011) briefed the symptoms of black rot caused by the aberrant strain of *P. multivesiculata.* Prominent initial symptoms include the formation of bleached water-soaked spots on leaves and pseudo-stems, which later turn black within 3–5 days. The premature drooping of infected leaves may also occur. In cases of severe rots in pseudo-stems and leaves, most of the infected plants eventually die. In general, this pathogen causes leaf blotches and rots of pseudobulbs/stems in seedlings or mature plants. However, no report of *Cymbidium* root and flower infection *by P. multivesiculata* was reported.

Needless to say, *Phytophthora* induces a variety of types of disease symptoms in infected orchid hosts, which are reported in various terms: seedling blight, root rot, top and shoot rot, heart and leaf rot, black rot, and crown rot, the end result of which is “the blackening of invaded parts and defoliation,” justifying the universally accepted term “Black rot of orchids” given by Rossetti (1943).

## Taxonomy of *Phytophthora* infecting orchid

Traditionally, all species of *Phytophthora* are known as Oomycetous fungi. They belong to the order Peronosporales, family Pythiaceae, class Oomycetes, and phylum Oomycota under the Kingdom “Fungi.” Currently, with the advancement in molecular, chemical, and ultra-structural studies, species of *Phytophthora* are considered to be “fungal-like organisms” or “pseudo fungi” with characteristics that are closer to the “heterokont,” biflagellate algae. They are chlorophyll-deficient “colourless algae.” According to the present concept, five important unique characteristics differentiate the Oomycota as a distinct group of an organism that is closer to the heterokont algae “Chromista” (Rossman and Palm, 2006). These characteristics include (a) the diploid state of vegetative mycelium, (b) the presence of β-glucans and cellulose in the cell wall, (c) sexual reproduction by heterogametangia (i.e., by oogonium and antheridium), (d) asexual reproduction through the production of motile heterokonts and (e) mitochondria with tubular cristae. Therefore, these are shifted into the separate Kingdom “Chromista” (Agrios, 1997) or “Straminopila” (Alexopoulos et al., 1996) rather than the true fungi kingdom “Fungi.” The taxonomic information is available in the first published monograph, “*Phytophthora* Diseases Worldwide,” by Erwin and Ribeiro (1996). Based on multiple loci, a detailed phylogeny of the genus *Phytophthora* was given by Blair et al. (2008). In addition, online taxonomic databases can be consulted using www.phytophthora.org for an instant requirement for *Phytophthora* identification (Park et al., 2008). Recently, Kroon et al. (2012) reported key details for the identification and delimitation of *Phytophthora* species, describing 10 clades in the genus *Phytophthora,* which accommodates 116 species, 15 of which still await valid publication.

## Characteristics and features of major *Phytophthora* species infecting orchid

### Phytophthora palmivora

This destructive pathogen was first identified as *Phytophthora omnivora* (Massee, 1899) and *Pythium palmivorum* (Butler, 1907) as the causal organisms of cocoa black pod disease and the destructive palm disease, respectively. Later on, both pathogens were merged into a single pathogen and renamed *Phytophthora palmivora* by Butler (1910). At present, it is globally accepted that *P. palmivora* causes black rot disease in both monopodial as well as sympodial orchids. According to the study of Hong et al. (1998), *P. palmivora* infecting *Cymbidium* produces uniform hyaline hyphae, no hyphal swellings, readily form chlamydospores (32–48 μm) in the medium, sporangiophores sympodially branched or sometimes terminal, zoosporangia form readily on agar and in water, zoosporangia conspicuously papillate, ellipsoid to ovoid (Erwin and Ribeiro, 1996; Hong et al., 1998), size 36–80 × 26–40 μm (average 57 × 34 μm), highly deciduous with short pedicel of 3–4 μm. The fungus can be characterized as heterothallic, oogonia globose, smooth 25–28 μm (average 26.5 μm) diameter with spherical oospores of 20–24 μm (average 22.4 μm) diameter, antheridia amphigynous, and oogonia aplerotic (Table 7). The optimum temperature for growth was 27–30^°^ C, including a minimum temperature of 8°C and a maximum temperature of 33°C.

**Table 7:** Characteristics of *Phytophthora* taxa infecting orchids.

*Phytophthora* sp.	Clade	Host	Infected tissue	Sex	A/Pg	Papillate	Author(s) and year
*Phytophthora nicotianae*	1	Polyphagous including many orchids hosts	Root/ foliage	He	A	P	Breda de Haan (1896)
*Phytophthora cactorum*	1b	Polyphagous including many orchids hosts	Root/ foliage	Ho	P	P	Schröter (1886)
*Phytophthora multivesiculata*	2	Specific on Cymbidium orchids	foliage	Ho	A	NP/SP	Ilieva et al. (1998)
*Phytophthora meadii*	2a	Polyphagous including *Vanilla* orchid host	Fruits /foliage/root	Ho	A	P	McRae (1918)
*Phytophthora capsici*	2b	Polyphagous including *Vanilla* host	Fruits/ foliage/root	He	A	P	Leonian (1922)
*Phytophthora palmivora*	4	Polyphagous including many orchid hosts	Flower/ foliage/root	He	A	P	Butler (1910)
*Phytophthora megasperma*	6b	Polyphagous including *Cymbidium* orchid host	roots	Ho	P	NP	Drechsler (1931)
*Phytophthora cinnamomi*	7b	Polyphagous including *Cymbidium* orchid host	Roots/foliage	He	A	NP	Rands (1922)
*Phytophthora erythroseptica*	8a	Polyphagous including *Cymbidium* orchid host	roots	Ho	A	NP	Pethybridge (1913)
*Phytophthora citricola*	2	Polyphagous including *Pleione* orchid host	Foliage/roots/whole plant	Ho	P	SP	Sawada (1927)
*Phytophthora. tropicalis*	2b	Polyphagous including *Vanilla* orchid host	Foliage/root/vine	He	A	P	Aragaki and Uchida (2001)

He = heterothallic, Ho = Homothallic, p = papillate, NP = non-papillate, SP = semi-papillate.

**Figure 7:**
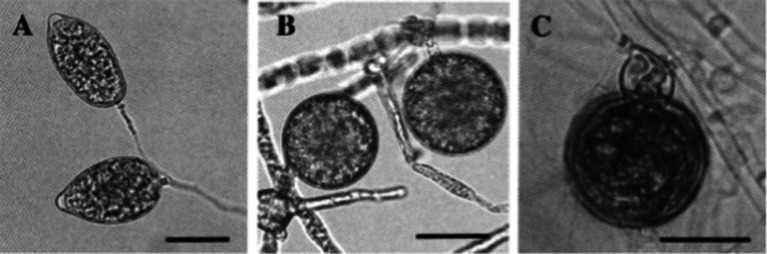
Morphological characteristics of *Phytophthora palmivora* on *Dinema polybulbon* orchid. **(A)** Sporangia, **(B)** chlamydospores, and **(C)** oospore (bar = 30 μm; Courtesy: Suzuki et al., 2008).

In Taiwan, the *Cattleya* black rot pathogen *P. palmivora* was studied by Yeh et al. (1998). According to their observation, the basic characteristics of this isolate were similar to those of *P. palmivora* infecting *Cymbidium* in the Republic of Korea. However, it was noted that the size of asexual (sporangia size 44.3–51.0 × 26.1–29.7 μm, with L/W ratios 1.61–1.75) and sexual structures (oogonia 23.9–32.2 μm diameter; oospores 20.3–27.6 μm diameter) were slightly different, but within the standard size of typical *P. palmivora.* The optimum, minimum, and maximum temperature requirements for mycelial growth were 24–32, 10, and 35°C, respectively. All host species of *P. palmivora* share similarities in basic distinguishing characteristics, but the size of various asexual and sexual structures may vary with isolates of different host species (Brasier and Griffin, 1979; Figure 7). A range of various morphological parameters for isolates of different hosts can be observed in “*Phytophthora* Diseases Worldwide” (Erwin and Ribeiro, 1996). Phylogenetically, *P. palmivora* belongs to clade 4 (Kroon et al., 2012; Martin et al., 2012).

### Phytophthora nicotianae

**Figure 8:**
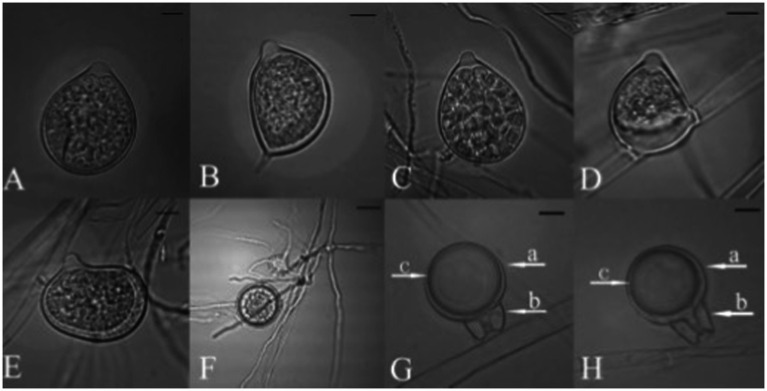
Morphological characteristics of *Phytophthora nicotianae*, **(A–E)** Papillate Sporangia, **(F)** Chlamydospore, **(G,H)** spherical oospore, a: Oogonia, b: antheridia (bar = 10 μm; Courtesy: Tao et al., 2011b).

The nomenclature of *P. nicotianae* has been under debate for a long time. Based on the detailed comparative morphological studies, Ho and Jong (1989) opined that *P. nicotianae* and *P. parasitica* should be considered single species. Following the International Code of Botanical Nomenclature, *P. nicotianae*, which has priority, should be used as the correct name throughout the world. Over the past few decades, the binomial name *P. nicotianae* has gained official recognition. The fungal colonies show cottony growth on V_8_A, CMA, and OMA with non-septate hyphae, hyaline (av. 5.05 μm wide), and no hyphal swellings. It consists of rounded, thick-walled rounded chlamydospores, either intercalary or terminal, measuring 19.71–49.58 μm (average diameter 30.15 μm). Sporangia are non-deciduous, terminal or occasionally intercalary, and conspicuously papillate; shapes may be different, such as pyriform, obpyriform, ellipsoid, ovoid, or spherical, measuring 23.07–70.31 × 17.09–57.91 μm (av. 46.20 × 34.89 μm) with mean L/W ratios of 1.34. Antheridia are amphigynous (Table 7) and round to short cylindrical; oogonia are smooth and globose, measuring 17.38–31.03 μm (av. 24.04 μm) in diameter (Figure 8). No antheridia or oogonia production is observed in single cultures until provided with appropriate mating strains (Tao et al., 2011b). *P. nicotianae* is a clade 1 *Phytophthora* species (Kroon et al., 2012).

### Phytophthora cactorum

This fungus is also one of the causal pathogens of black rot in orchids. It mainly occurs in temperate regions. *P. cactorum* was first described as a pathogen of cactus in 1870. Since then, it has been reported on several plant species worldwide, infecting approximately 150 plant genera belonging to at least 250 known plant species (Erwin and Ribeiro, 1996). For decades, it has been well-established as an orchid pathogen in many temperate countries. *P. cactorum* can proliferate on a range of standard laboratory mediums, including PDA, CMA, LBA, and V8A. PDA and V8A show white fungal colonies. Mycelium is hyaline, coenocytic, and readily develops zoosporangia on culture media or host tissue maintained in a humidity chamber under constant fluorescent light at 25–28°C (Cating et al., 2010). Zoosporangia is typically ellipsoidal to pear-shaped to spherical (size 30 × 26 μm), usually terminal, papillate, easily detachable, and pedicel less than 4 μm long. Owing to the homothallic nature of the fungus, it is easy for it to develop sexual structures on standard laboratory media (Erwin and Ribeiro, 1996; Gallegly and Hong, 2008). Antheridia are paragynous (Table 7), spherical to irregularly clavate, and measure 13 × 15 (−21) μm. Oogonia are spherical or taper at the base and measure 25–32 μm in diameter. Oospores have an aplerotic shape with a diameter of 20–26 μm. Chlamydospores are usually not formed; however, they may be terminal or intercalary, measuring 20–40 μm in diameter if produced. *P. cactorum* is a clade 1b *Phytophthora* species (Kroon et al., 2012).

### Phytophthora multivesiculata

**Figure 9:**
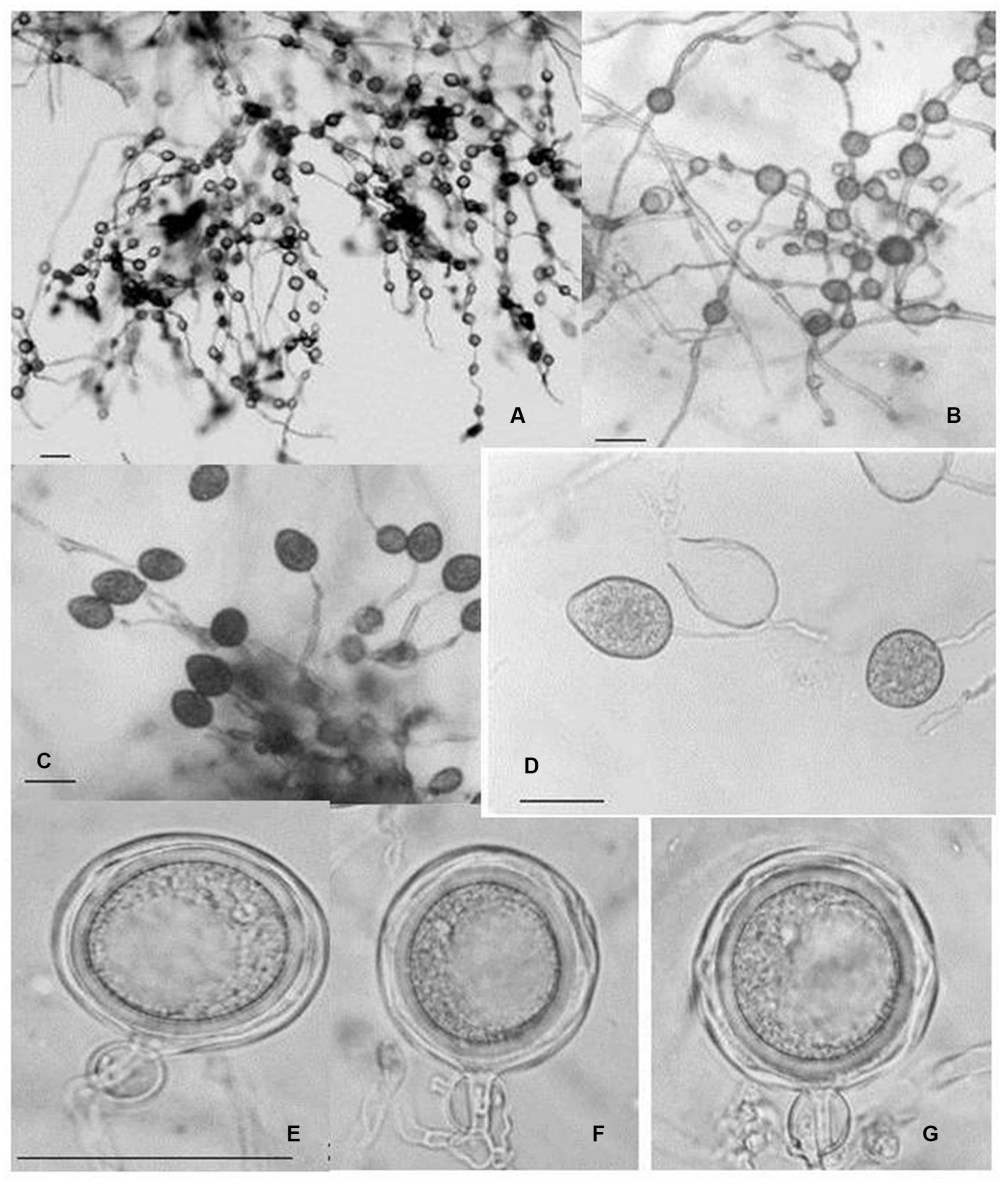
Morphological characteristics of *P. multivesiculata* infecting Cymbidium hybridum: **(A, B)** hyphal swellings in chain; **(C)** primary sporangia, **(D)** proliferation of Sporangia; **(E–G)** oospores (bar = 50 mm; Courtesy: Chern et al., 2011).

*Phytophthora multivesiculata* has been reported as a causal agent of leaf blotch and rots of pseudobulbs in *Cymbidium*. The disease was first reported by Ilieva et al. (1998) with the speciation of this fungus under the genus *Phytophthora*. Colonies of *P. multivesiculata* on V8, PDA, OA, and CA show moderately fluffy aerial mycelium, a bit denser in the center of the colony on PDA; on CMA, mycelium is submerged (Ilieva et al., 1998). In CMA, the major hyphae are coenocytic, branched, and 6 mm wide; the hyphal swellings are distinctive characteristics of the fungus (Figure 9). Numerous hyphal swellings were common on agar and in water culture. Hyphal swelling is usually described as spherical, elliptical, catenulate, and grouped. Hyphal swellings typically vary from 14 to 36 mm in diameter, new branches develop at sharp angles, and no chlamydospores were reported. The fungus is reported to be homothallic, as both asexual and sexual productions are observed. Sexual structures are produced on both agar and liquid media as well as in plant tissues. Sporangiophores are usually long, slim (2.0–3.0 μm), mostly twisted, and borne singly on the sporangiophores. Sympodial arrangements of sporangia are also recorded occasionally (Ilieva et al., 1998). Sporangia are produced in both solid and liquid media, measuring 45.0 μm (30.0–60.0 μm) × 33.0 μm (20.0–41.0 μm). They may be ovoid, obpyriform, non-papillate, or semi-papillate, and exit pores range from 8.0 to 14.0 μm wide. Antheridia are amphigynous (95%), and some are diclinous (Table 7). It can be irregularly spherical or ellipsoidal. Oogonia are spherical and smooth-walled and vary from 28.0 to 50.0 μm on V8 medium. Oospores are mainly aplerotic and vary from 24.0 to 42.0 μm (Ilieva et al., 1998).

The characteristics of *P. multivesiculata* resemble the biological features of *P. porri* and *P. megasperma. H*owever, with critical examination, *P. multivesiculata* can be distinguished from *P. porri* and *P. megasperma* based on morphology, pathogenicity, and temperature requirements (Figure 9). Although differences are very small, a clear-cut distinction prevails among these species. To date, *P. porri* is known to be pathogenic to the genus *Allium* and *Brassica,* but *P. multivesiculata* is pathogenic to the genus *Cymbidium*. *P. porri* has slow growth and low optimum and maximum growth temperatures. *P. multivesiculata* exhibits a considerably higher growth rate at 20°C than *P. porri*, but lower than those observed for *P. megasperma.* The maximum growth temperature for *P. multivesiculata* was 35°C. Typical catenulate hyphal swellings for *P. multivesiculata* may be observed occasionally in *P. Porri*, but their appearance is mainly in a more radiating manner. Hyphal swellings are more frequent and abundant in *P. multivesiculata* than in *P. megasperma*. Furthermore, antheridia in *P. multivesiculata* are mainly amphigynous (95%); they are mostly paragynous in *P. megasperma*. An aberrant strain of *P. multivesiculata* from Taiwan on *Cymbidium* was reported by Chern et al. (2011). Chain-like catenulate hyphal swellings of Taiwanese isolates were similar to those of Netherlands isolates of *P. multivesiculata*. However, sporangia, oogonia, and oospores are larger in the Taiwanese isolates than in the Netherlands isolates. The oogonial wall of Taiwanese isolates is echinulate, but Netherlands isolates have smooth wall oogonia. In addition, the maximum growth temperature for the Taiwanese isolate of *P. multivesiculata* is 29°C, whereas the maximum growth temperature for the Netherlands isolates is 35°C. *P. multivesiculata* exhibits isozyme profiles that differ from the profiles of *P. megasperma* and *P. porri* types (Ilieva et al., 1998). *P. multivesiculata* belongs to a clade 2 *Phytophthora* species (Kroon et al., 2012).

## Phylogenetic relationship among orchid infecting *Phytophthora* species

**Figures 10:**
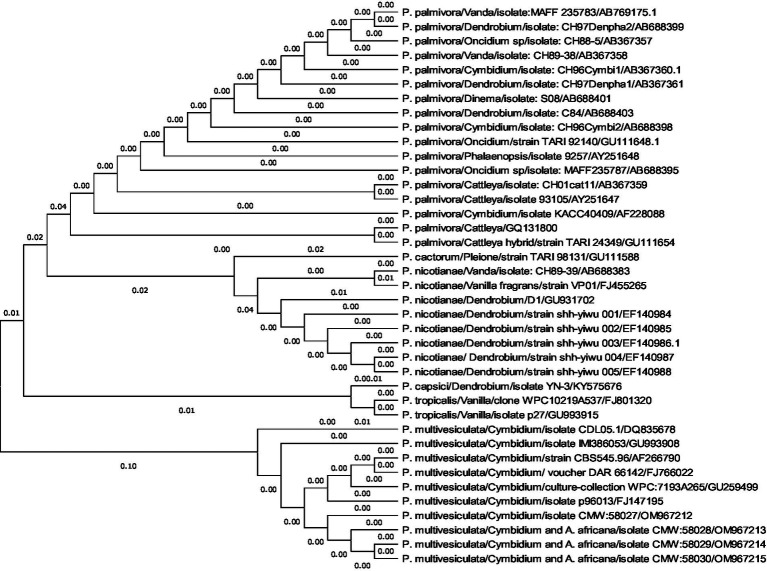

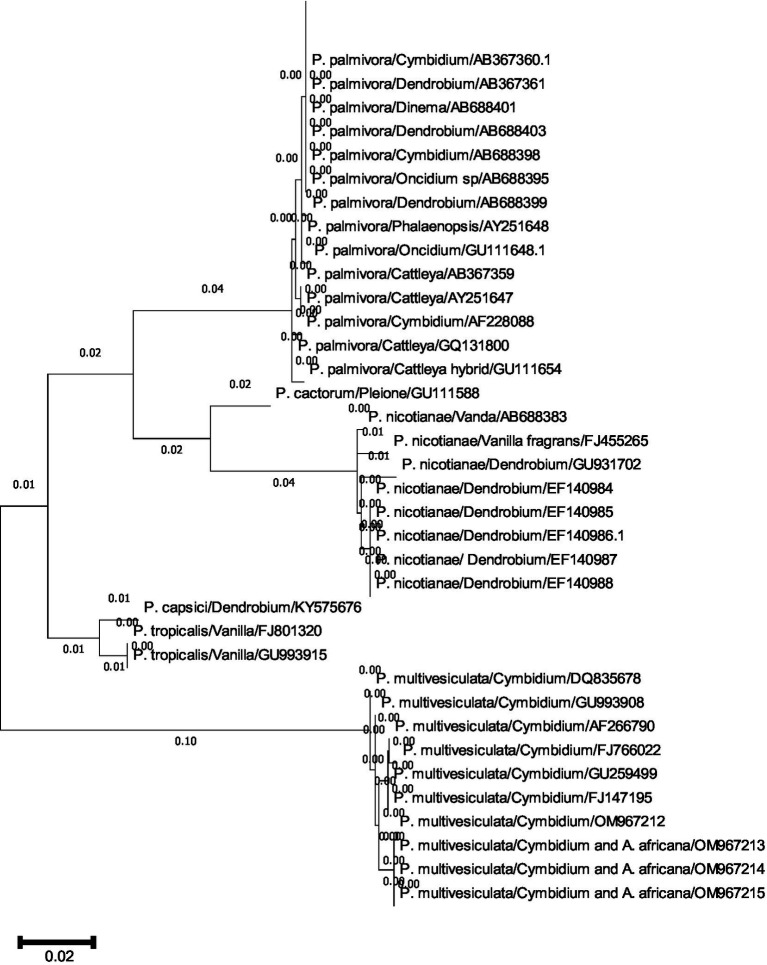
**(A,B)** Evolutionary analysis by maximum likelihood method. **(A)** Phylogenetic relationship of different species of *Phytophthora* infecting orchids. **(B)** Phylogenetic relationship of different species of *Phytophthora* infecting orchids.

A comprehensive phylogenetic analysis of *Phytophthora* species comprising 39 sequences of 6 notable orchid-infecting species such as *P. palmivora, P. cactorum, P. nicotianae, P. capsici, P. tropicalis*, and *P. multivesiculata* was carried out, which exhibited diverse host affinities toward orchids such as *Vanda, Dendrobium, Oncidium, Cymbidium, Phalaenopsis, Cattleya, Pleione,* and *Vanilla*. The investigation led to the emergence of four distinct clades, as visually depicted in Figures 10A,B. Remarkably, *P. multivesiculata* was observed to cluster within clade I, while *P. palmivora* species formed a cohesive group in clade IV. Additionally, the study unveiled a close relationship between *P. tropicalis* and *P. capsici* within clade II and a clustering of *P. cactorum* with *P. nicotianae* in clade III. These findings contribute valuable insights into the evolutionary dynamics and relatedness among the *Phytophthora* species infecting orchids studied.

The evolutionary history was inferred by using the maximum likelihood method and the Tamura–Nei model (Tamura and Nei, 1993). The tree with the highest log-likelihood (−2779.26) is shown. The initial tree(s) for the heuristic search were obtained automatically by applying neighbor-join and BioNJ algorithms to a matrix of pairwise distances estimated using the Tamura–Nei model and then selecting the topology with a superior log-likelihood value. This analysis involved 39 nucleotide sequences. Codon positions included were 1st + 2nd + 3rd + Non-coding. There were a total of 964 positions in the final dataset. Evolutionary analyses were conducted in MEGA11 (Tamura et al., 2021).

### Mating types and races of *Phytophthora* infecting orchids

The production of oospores in *Phytophthora* is very important from an evolutionary point of view for pathogenic variability, fitness of the conventional hosts, and their constantly changing patterns with the continuous evolution of new man-made hybrids. In some of the species of *Phytophthora* (called heterothallic), two opposite sexual types are prerequisite for the formation of oospores through sexual reproduction. Other groups of *Phytophthora* (called homothallic) do not require this specificity, but they can produce oospores themselves. Based on the production of oospores in paired cultures, “mating type” or “compatibility type” A^1^ and A^2^ in *Phytophthora* were first conceptualized by Gallegly and Galindo (1958) using the potato-*Phytophthora infestans* pathosystem. It is globally used for all *Phytophthora* species, which consist of only two mating types. After 30 years, another mating type, “A^1^A^2^,” was introduced by Ko (1988); therefore, there are three mating types, A^1^, A^2^, and A^1^A^2^, in the genus *Phytophthora* at present.

Among the *Phytophthora* species infecting orchids, *P. palmivora, P. nicotianae, P. cinnamomi,* and *P. capsici* are heterothallic species, and so they can multiply asexually as well as sexually (Erwin and Ribeiro, 1996). In the presence of both mating types, sexual reproduction leads to the production of oospores, which ensures a longer period of natural overwintering in addition to genetic variability in the germination of oospores, making races or pathotypes of the pathogen more complex. Both mating types A1 and A2 (Table 8) have been reported for *P. palmivora* infecting various orchid genera in French Polynesia (Mu and Tsao, 1987); Hawaii, USA (Ashby, 1929b; Uchida and Aragaki, 1991); Ceylon (Sri Lanka; Ashby, 1929b); Java (Ashby, 1922, 1929a); and Taiwan (Ho, 1990; Yeh et al., 1998). However, only the A1 mating type was reported from the Republic of Korea (Hong et al., 1998), Sri Lanka (Ashby, 1922, 1929a), and Japan (Suzuki et al., 2008).

**Table 8:** Mating type distribution of *Phytophthora* species infecting various orchid genera.

Sl. no.	*Phytophthora* sp.	Orchid host	Country	Mating type		References
1.	*P. capsici*	*Vanilla* sp.	Indonesia	A1	-	Tombe and Liew (2010)
2.	*P. nicotianae*	*Cattleya* sp.	Taiwan	A^1^	-	Ho (1990)
3.	*P. nicotianae*	*Dendrobium phalaenopsis*	Taiwan	-	A^2^	Ho (1990)
4.	*P. nicotianae*	*Dendrobium* sp.	Taiwan	-	A^2^	Ho (1990)
5.	*P. nicotianae*	*Phalaenopsis* sp.	Taiwan	A^1^	A^2^	Ho (1990)
6.	*P. nicotianae*	*Cymbidium sinense*	Taiwan		A^2^	Ann (1995)
7.	*P. nicotianae*	*Cymbidium rubrigemmum*	Taiwan		A^2^	Ann (1995)
8.	*P. nicotianae*	*Cymbidium ensifolium*	Taiwan		A^2^	Ann (1995)
9.	*P. nicotianae*	*Vanilla* sp.	French Polynesia	A^1^	A^2^	Mu and Tsao (1987)
10.	*P. nicotianae*	*Dendrobium candidum*	China	-	A^2^	Zhang et al. (2006)
11.	*P. nicotianae*	*Cattleya* sp.	Florida, USA	-	A^2^	Patel et al., (2016)
12.	*P. nicotianae*	*Oncidium* sp.	Florida, USA	A^1^	-	Patel et al. (2016)
13.	*P. palmivora*	*Dinema polybulbon*	Japan	A^1^	-	Suzuki et al. (2008)
14.	*P. palmivora*	orchids	Hawaii, USA	A^1^	A^2^	Uchida and Aragaki (1991)
15.	*P. palmivora*	*Vanda* sp.	Hawaii, USA	A^1^	A^2^	Ashby (1929b)
16.	*P. palmivora*	*Dendrobium* sp.	Ceylon (Sri Lanka)	A^1^	-	Ashby (1922, 1929a)
17.	*P. palmivora*	*Cattleya* sp.	Ceylon (Sri Lanka)	A^1^	A^2^	Ashby (1929b)
18.	*P. palmivora*	*Vanda* sp.	Java (Indonesia)	A^1^	A^2^	Ashby (1922, 1929a)
19.	*P. palmivora*	*Cattleya* sp.	Java (Indonesia)	A^1^	A^2^	Ashby (1922, 1929a)
20.	*P. palmivora*	*Phalaenopsis* sp.	Taiwan	A^1^	A^2^	Ho (1990)
21.	*P. palmivora*	*Cattleya* sp.	Taiwan	A^1^	A^2^	Yeh et al. (1998)
22.	*P. palmivora*	*Oncidium sp*	Taiwan	A^1^	-	Ann (1995)
23.	*P. palmivora*	*Paphiopedilum* sp.	Taiwan	A1		Ann (1995)
24.	*P. palmivora*	*Phaius sp*	Taiwan	A1		Ann (1995)
25.	*P. palmivora*	*Cymbidium oiwakensis*	Taiwan	A^1^		Ann (1995)
26.	*P. palmivora*	*Cymbidium formosanum*	Taiwan	A^1^		Ann (1995)
27.	*P. palmivora*	*Vanilla* sp.	French Polynesia	A^1^	A^2^	Mu and Tsao (1987)
28.	*P. palmivora*	*Dendrobium phalaenopsis*	Japan	-	A2	Masanto et al. (2019)
29.	*P. palmivora*	*Cymbidium*	Japan	A^1^	-	Masanto et al. (2019)
30.	*P. palmivora*	*Cattleya*	Japan	A1	-	Masanto et al. (2019)
31.	*P. palmivora*	*Vanda*	Japan	A^1^	-	Masanto et al. (2019)
32.	*P. palmivora*	*Oncidium*	Japan	-	A2	Masanto et al. (2019)
33.	*Phytophthora palmivora*	*Cymbidium* sp.	Republic of Korea	A^1^	-	Hong et al. (1998)

Both mating types A1 and A2 were recorded for *P. nicotianae* in Taiwan (Ho, 1990) and French Polynesia (Mu and Tsao, 1987), infecting *Phalaenopsis* and *Vanilla* orchids, respectively, and other *P. nicotianae* isolates were either A1 or A2 in Taiwan and Florida (Patel et al., 2016). Reports of races that are common in heterothallic species of *Phytophthora, viz.*, 24 races in *P. capsici* causing pepper root rot (Barchenger et al., 2018), are in the public domain. Interestingly, no races of *P. palmivora, P. nicotianae*, or *P. capsici* infecting orchids have been reported globally to date, as per our knowledge. One reason could be that sufficient scientific attention was not paid to this aspect with special reference to orchido-phytopathogenic *P. palmivora, P. nicotianae*, and *P. capsici* on various species or hybrids of the orchid world over. Another reason could be that the genomic organization of present-day interspecific, intergeneric, and/or multi-generic hybrids is so complex that the successful establishment of differential lines/ hosts of orchids (based on R-gene) for race identification may not be possible or is a researchable issue for future attention.

### Epidemiology of *Phytophthora* species infecting orchids

The epidemiological aspects of the species of *Phytophthora* infecting orchids have not been studied well under field conditions, unlike the other *Phytophthora* species infecting commercially cultivated fields and horticultural crops. Most of the available literature on epidemiology is based only on *in vitro* observations. Perhaps Hine (1962) was the first scientist to study the influence of temperature and length of wetness period on the mycelial growth, zoosporangia, and zoospore formation in *P. palmivora* infecting *Vanda*, *Cattleya*, *Epidendrum*, and *Dendrobium* in Hawaii. The minimum and optimum temperatures for mycelial growth on V_8_A medium were observed to be 10°C and 28–31°C, respectively. The fungus can grow up to 25°C but not at 37°C. The zoosporangia and chlamydospore formation start after 3 days on V_8_A medium and stems or leaves of *Vanda* orchids at a temperature between 20 and 31°C. Zoospore formation requires optimum water temperatures between 15 and 25°C and remains active for the longest time (5 h) in water at 25°C. No zoospores are visible at a water temperature of 28°C. In *Vanda*, wounded leaves can be infected with zoospores at temperatures between 15 and 31°C. Generally, the disease does not occur during the hotter months of the year, even if there is sufficient moisture on the plant surface because of the inhibition of zoospore formation above 25°C. According to Yeh et al. (1998), the minimum, optimum, and maximum temperatures for mycelial growth of *P. palmivora* causing black rot of *Vanda* are 10, 24–32, and 35°C, respectively. The optimum temperature requirement for zoosporangia production on V_8_A and the leaf surface of *Cattleya* is 24°C. The maximum number of zoosporangia is produced at 100% RH, whereas no zoosporangia are produced below 80% RH. Zoosporangia germinate directly at an optimum temperature of 24°C whereas indirect germination of zoosporangia can occur from 8 to 32°C with an optimum temperature of 16°C. No zoospores are formed at 35°C.

A comparative study was conducted by Hsieh (1984) on the temperature requirements for zoosporangia formation and germination and chlamydospore formation in *P. palmivora* and *P. nicotianae*. Both produce zoosporangia between 12 and 32°C (optimum at 24–28°C for *P. palmivora* and 24°C for *P. nicotianae*), and a large number of zoosporangia are formed at 95% RH but no zoosporangia are formed at 85% RH. Both pathogens produce abundant zoosporangia at 90–100% RH on the disease leaves of orchids. They prefer to produce more zoosporangia on diseased leaves (such as *Vanda* sp.) than chlamydospores at 90–95% RH; however, at a lower range of RH of 80–85% and a higher temperature of 36°C, only chlamydospores are produced. Young zoosporangia germinate indirectly between 8 and 28°C, and mature zoosporangia germinate directly between 20 and 28°C. Chlamydospore germination takes place at a temperature range of 8–37°C, with an optimum temperature of 28 and 24°C for *P. palmivora* and *P. nicotianae,* respectively. They also observed that a favorable temperature for disease development is 16–36°C, with an optimum of 28°C. At the saturated condition of relative humidity, lesion development was faster but decreased in drier conditions on the diseased leaves of orchids.

*Phytophthora multivesiculata* is host-specific to the *Cymbidium* orchid. However, epidemiological data on this pathogen in orchids are limited. *P. multivesiculata* on *Cymbidium* in the Netherlands was reported by Ilieva et al. (1998). They observed that the disease occurs on the leaves after prolonged periods of rain or on the indoor plant after irrigation. *P. multivesiculata* grows at a faster rate on the V_8_A medium at 20°C, and the maximum temperature for growth is 25°C (Ilieva et al., 1998). The temperature range for growth of an atypical (aberrant strain) *P. multivesiculata* that causes black rot on *Cymbidium* species is reported to be 10–29°C in Taiwan, and there is practically no growth at 30°C, which is 6°C less than that of typical *P. multivesiculata* (with 35°C as the highest growth temperature) described from the Netherland. The optimum growth temperature of Taiwanese isolate is reported to be 24°C on a V_8_A medium (Chern et al., 2011).

### Dissemination of orchids infecting *Phytophthora*

**Figure 11:**
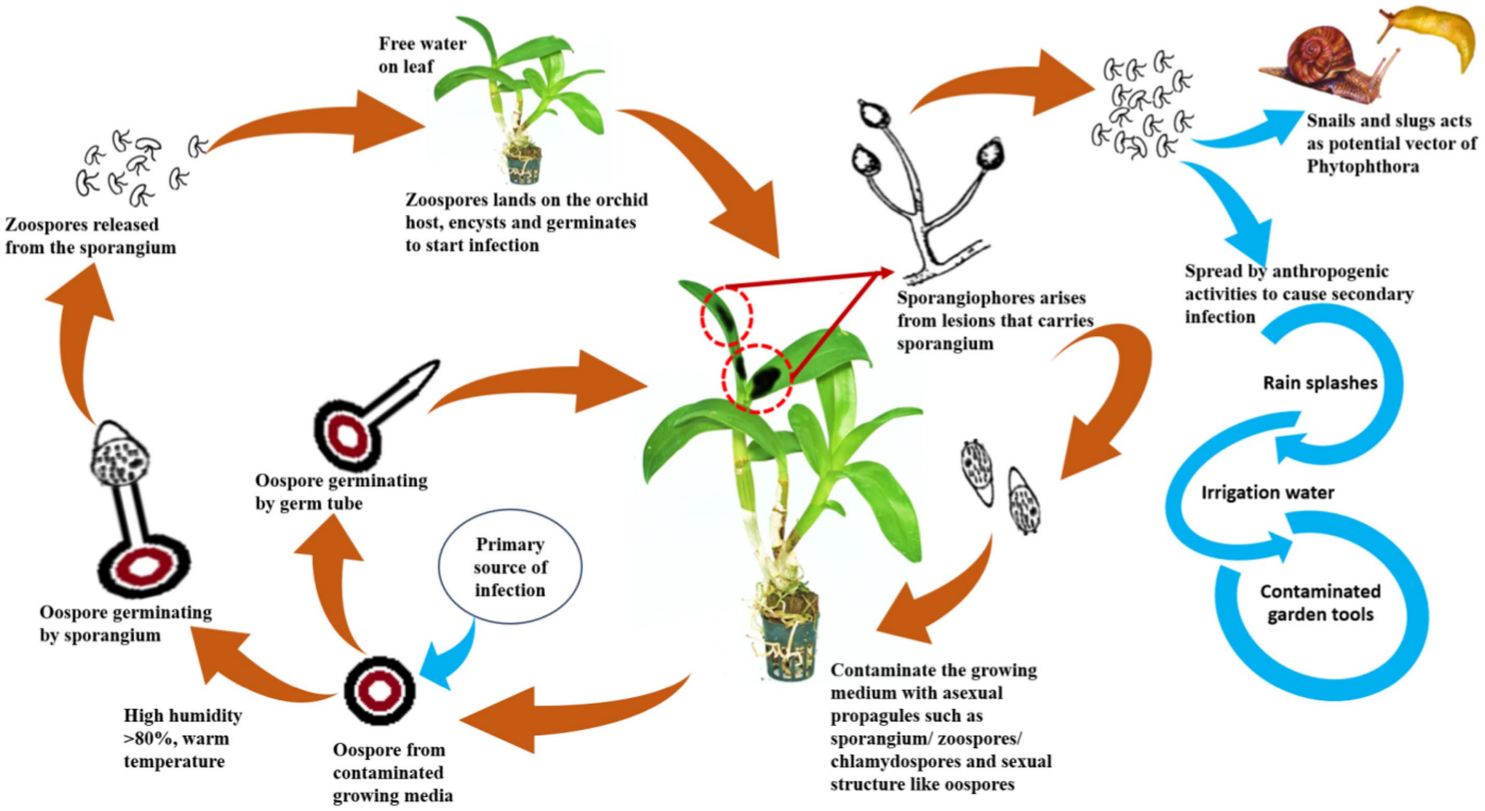
Disease cycle of *Phytophthora* spp.

To develop management strategies, it is crucial to understand the dispersal of plant pathogens, particularly water molds. Since most of the orchids are grown under controlled conditions in artificially constructed polyhouses/net houses, anthropogenic factors are mostly contributed to the spread or dissemination of orchid *Phytophthora* diseases. The spores may spread from one plant to another neighboring plant by watering or water splashing; thus, irrigation water can act as a medium for the spread of zoospores (Uchida, 1994). *P. palmivora* is reported to be spread by sprinkling water and by the import of young plant materials into greenhouse crops (Orlikowski et al., 2008). Contaminated garden tools, agricultural implements, plant transport trailers, or carts can also passively spread *Phytophthora*. Snails and slugs are also considered potential agents for the spread of *Phytophthora* zoospores by either carrying the zoospores on their bodies or by ingesting the zoospores and later discharging the viable zoospores through excreta on the plant surface (Uchida and Aragaki, 1991; Figure 11). The intercontinental movement of planting material can passively disseminate *Phytophthora*. It was observed that imported prefinished *Cattleya* liners from Thailand are often infected with *P. cactorum* during monsoon seasons in Florida, USA (McMillan et al., 2010).

### Molecular diagnostic for major orchids infecting *Phytophthora*

Orchids are legally or illegally traded across the world (Fay, 2015), due to which live orchid plant materials may carry *Phytophthora* either on plant surfaces or in potting media from one country to another, where it may or may not be present as a serious pathogen. Hence, the diagnosis of orchid diseases caused by *Phytophthora* species is of paramount importance for advocating an appropriate management strategy and regulation of the movement of this destructive phytopathogen through traded orchid materials. Proper diagnosis of any *Phytophthora* species infecting orchids depends on the pathogenicity and morphology of the pathogenic fungi or through molecular techniques. Symptoms, signs, and pathogenicity tests of isolated fungi give vital clues about the hosts, but these cannot always provide the true identity of the *Phytophthora* species. The reality is that the diagnosis of orchid *Phytophthora* based on symptomatic observation is hard and difficult to distinguish from those caused by the phytobacterial orchid pathogen *Erwinia carotovora* subsp. *carotovora* (Su and Leu, 1992), and possibly it becomes worse because these orchid pathogens may simultaneously infect orchids (Tsai et al., 2006).

Traditionally, the identification of the orchid *Phytophthora* is based on the characteristics of mycelium, hyphal swellings, branching of sporangiophores, shape and size of zoosporangia, presence of chlamydospores with their size and position, formation of antheridia, and oogonia along with their position (either paragynous or amphigynous) and oospores characters that confirm the fungi up to the level of the genus “*Phytophthora*.” Diagnostic tools used certainly depend on the ease of available facilities at the diagnostic laboratory or research units. Prior to the development of advanced diagnostic molecular techniques, most mycologists, or specifically plant pathologists, used conventional techniques of culturing the fungi on specific media for sporulation or fructification, followed by microscopic observation to define the fungi up to species level using identification keys given by stalwart mycologists’ time to time. Often, identification based on taxonomic keys leads to erroneous identification when compared with molecular data.

Therefore, nowadays molecular diagnostic data with multiple parameters are thought to be essential for the correct identification of the *Phytophthora* species infecting orchids. Sequencing the internal transcribed spacer (ITS) regions of rDNA is conventionally used as a molecular diagnostic tool to distinguish between *P. cactorum, P. palmivora*, and *P. nicotianae*, as well as many other *Phytophthora* species (Brasier and Griffin, 1979; Cooke and Duncan, 1997; Cooke et al., 2000). Based on the sequence analysis of rDNA-ITS regions, *P. palmivora* causes black rot of *Dinema polybulbon* in Japan (Suzuki et al., 2008), *P. nicotianae* causing *Dendrobium candidum* blight in China (Zhang et al., 2006), and *P. multivesiculata* causing leaf blotch and rot on *Cymbidium* orchids in New Zealand (Hill, 2004) have been identified (Figure 7).

However, the variation in DNA sequence in the ITS regions may not be enough to discriminate among closely related *Phytophthora* species (Martin et al., 2014), so it essentially necessitates the use of multiple criteria for species identification. Sequencings of the ITS1, 5.8S rRNA gene, and ITS2 regions of a *Phytophthora* isolate were carried out to identify *P. palmivora* causing black rot on *Cattleya* orchids in Florida (Cating et al., 2010). *P. nicotianae* causing blight on *Dendrobium aurantiacum, Dendrobium chrysanthum*, *Dendrobium chrysotoxum,* and *Dendrobium thyrsiflorum* was confirmed by using multiple molecular data along with morphology and pathogenicity tests in Yunnan Province, China. The gene sequences targeting multiple genes such as ITS1, 5.8S rRNA, ITS2, and β-tubulin of the pathogen were analyzed and compared to those of other known *P. nicotianae* available on GenBank. The results were used to confirm the identification of the orchid pathogen, *P. nicotianae* infecting different *Dendrobium* species (Tao et al., 2011b).

Isozyme analysis has proven to be a powerful tool in *Phytophthora* taxonomy (Oudemans and Coffey, 1991a,b). Isozyme analysis, along with the ITS sequence of a suspected *Phytophthora* isolate, can provide further insights into the pathogen for species differentiation among closely related *Phytophthora* species. Profiles of isozymes such as MDH, i.e., malate dehydrogenase; MDHP, i.e., a malic enzyme; and isocitrate dehydrogenase (IDH) of *Phytophthora* sp. infecting *Cymbidium* orchids and closely related *P. porri* and *P. megasperma* were analyzed, and it was found that the IDH pattern of *Phytophthora* sp. infecting *Cymbidium* is different from those of *P. porri* and *P. megasperma.* Furthermore, the ITS sequence of *Phytophthora* sp. causing blackening of leaves and stems on *Cymbidium* orchids in the Netherlands has been determined as *P. multivesiculata,* which was found to be unique but closely related to that of *P. citricola*. However, *P. multivesiculata* has amphigynous antheridia and hyphal swellings, whereas *P. citricola* has paragynous antheridia and no hyphal swellings. Thus, *P. multivesiculata,* infecting *Cymbidium* orchids, was identified as a new species of *Phytophthora* (Ilieva et al., 1998). An aberrant strain of *Phytophthora* sp. causing black rot of *Cymbidium* orchids in Taiwan was precisely identified as *P. multivesiculata* based on analysis of soluble protein patterns closely related to *Phytophthora* species and the sequence of ITS regions, including ITS1-5.8S rDNA-ITS2 plus, partial 18S rRNA and 28S rRNA of *Phytophthora* sp. infecting *Cymbidium* orchids (Chern et al., 2011).

In the era of global trade of orchids, quick and faster detection techniques are in great demand, particularly in quarantine stations in airports or land frontiers or in plant disease diagnostic laboratories, where the diagnosis of *Phytophthora* diseases in orchids is carried out. Based on the traditional method of isolation in pure culture of *Phytophthora* from disease plants, followed by identification based on morphology, this is indeed laborious and time-consuming. A rapid and simple process of diagnosis, i.e., nested PCR assay, has been developed for fast and accurate detection of orchid *Phytophthora* pathogens at the National Taiwan University, Taiwan (Tsai et al., 2006). In this unique diagnostic technique, fungal DNA is directly extracted from infected orchid (*Oncidium* sp.) host tissue, which is then subjected to PCR amplification with a *Phytophthora*-specific primer set. Amplification of DNA fragments of approximately 1 kb confirms the presence of *Phytophthora* pathogens in the infected orchid sample. Subsequently, nested PCR is run to identify the species of *Phytophthora* using the amplified product of the first PCR as the template DNA and species-specific oligonucleotides as the species primers (Pal1s/Pal2a for *P. palmivora* and Paris/Par2a for *P. parasitica* = *nicotianae*). Amplification of specific DNA fragments indicates the presence of either *P. palmivora or P. nicotianae* or both in the infected orchid host (Tsai et al., 2006). Nested PCR assay, indeed, offers sensitive and rapid detection of orchid *Phytophthora* pathogens. If properly designed, this assay can also be used to detect *Phytophthora* pathogens from contaminated potting media of orchids and thereby greatly contribute to the early diagnosis of destructive *Phytophthora* diseases of orchids. Various sequence-based PCR techniques along with specific primers are available for the detection of *Phytophthora* spp. infecting orchids (Table 9) and they can be used suitably for the diagnosis of *Phytophthora* diseases of orchids.

**Table 9:** Sequence-based PCR detection of different *Phytophthora* species infecting orchids with primer pairs.

Pathogen identified	Orchid host	Locus	Forward primer name	Forward primer sequence	Reverse primer name	Reverse primer sequence	Reference
*Phytophthora nicotianae*	*Dendrobium*	*ITS*	ITS6	5′-GAAGGTGAAGTCGTAACAAGG-3′	ITS4	5′-TCCTCCGCTTATTGATATGC-3′	Zhang et al. (2006)
	*Dendrobium*	*ITS*	ITS1	5′-TCCGTAGGTGAACCTGCGG-3′	ITS2	5′-GCTGCGTTCTTCATCGATGC-3′	Tao et al. (2011a)
	*Dendrobium*	*Btub*	tubuF2	5′-ACGGCTCGAGGATGACCATG-3′	TubuR1	5′-CCTGGTACTGCTGGTACTCAG-3′	Tao et al. (2011a)
	*Vanilla*	*ITS*	ITS1	5′-TCCGTAGGTGAACCTGCGG-3′	ITS4	5′-TCCTCCGCTTATTGATATGC-3′	Liu et al. (2008)
*Phytophthora* multivesiculata	*Cymbidium* and *Ansellia*	*COXI*	FM84	5′-TTT AAT TTT TAG TGC TTT TGC-3′	FM83	5′-CTCCAATAAAAAATAACCAAAAATG-3′	Bose and Hammerbacher (2022) (Multiple gene test)
	*Cymbidium* and *Ansellia*	*Btub*	tubuF1A	5′-ACGGCTCGAGGATGACCATG-3′	tubuR1	5′-CCTGGTACTGCTGGTACTCAG-3′	
	*Cymbidium* and *Ansellia*	*ITS*	DC6	5′-GAGGGACTTTTGGGTAATCA-3′	ITS4	5′-TCCTCCGCTTATTGATATGC-3′	
	*C. hybridum*	*ITS*	ITS4	5′-TCCTCCGCTTATTGATATGC-3′	ITS5	5′-GGAAGTAAAAGTCGTAACAAGG-3′	Chern et al. (2011)
*Phytophthora palmivora*	Cattleya	ITS1/ 28S	Phy1s	5′-ACTTTCCACGTGAACCGTATCA-3′	Phy2a	5′-GCA CGA GCC ACT CAG GGA TG-3′	Tsai et al. (2006) (Nested PCR)
		ITS	ITS1	5′-CACGTGAACCGTATCAAAACT-3′	ITS2	5′-CAA TCA TAC CAC CAC AGC TGA-3′	
	Cattleya	ITS	ITS5	5′-GGAAGTAAAAGTCGTAACAAGG-3′	ITS4	5′-TCCTCCGCTTATTGATATGC-3′	Cating et al. (2010)
	Dinema	ITS	ITS5	5′-GAAGTAAAAGTCGTAACAAGG-3′	ITS4	5′-CCTCCGCTTATTGATAGC-3′	Suzuki et al. (2008)
	Rhynchostylis	ITS	ITS5	5′-GAAGTAAAAGTCGTAACAAGG-3′	ITS4	5′-TCCTCCGCTTATTGATAGC-3′	Wongwan et al. (2021)
		*COXI*	OomCoxILevup	5′-TCAWCWMGATGGCTTTTTTCAAC-3′	Fm85mod	5′-RRHWACKTGACTDATRATACCAAA-3′	
*Phytophthora capsici*	Dendrobium	ITS	ITS1	5′-TCCGTAGGTGAACCTGCGG-3′	ITS4	5′-TCCTCCGCTTATTGATATGC-3′	Li et al. (2018)
		*CoxII*	FM75F	5′-CCTTGGCAATTAGGATTTCAAGAT-3′	FM78R	5′-CAAATTTCACTACATTGTCC-3′	
*Phytophthora cactorum*	Pleione	ITS	ITS1	5′-CACGTGAACCGTATCAAAACT-3′	ITS2	5′-CAA TCA TAC CAC CAC AGC TGA-3′	NCBI database

## Management strategies and options

**Figure 12:**
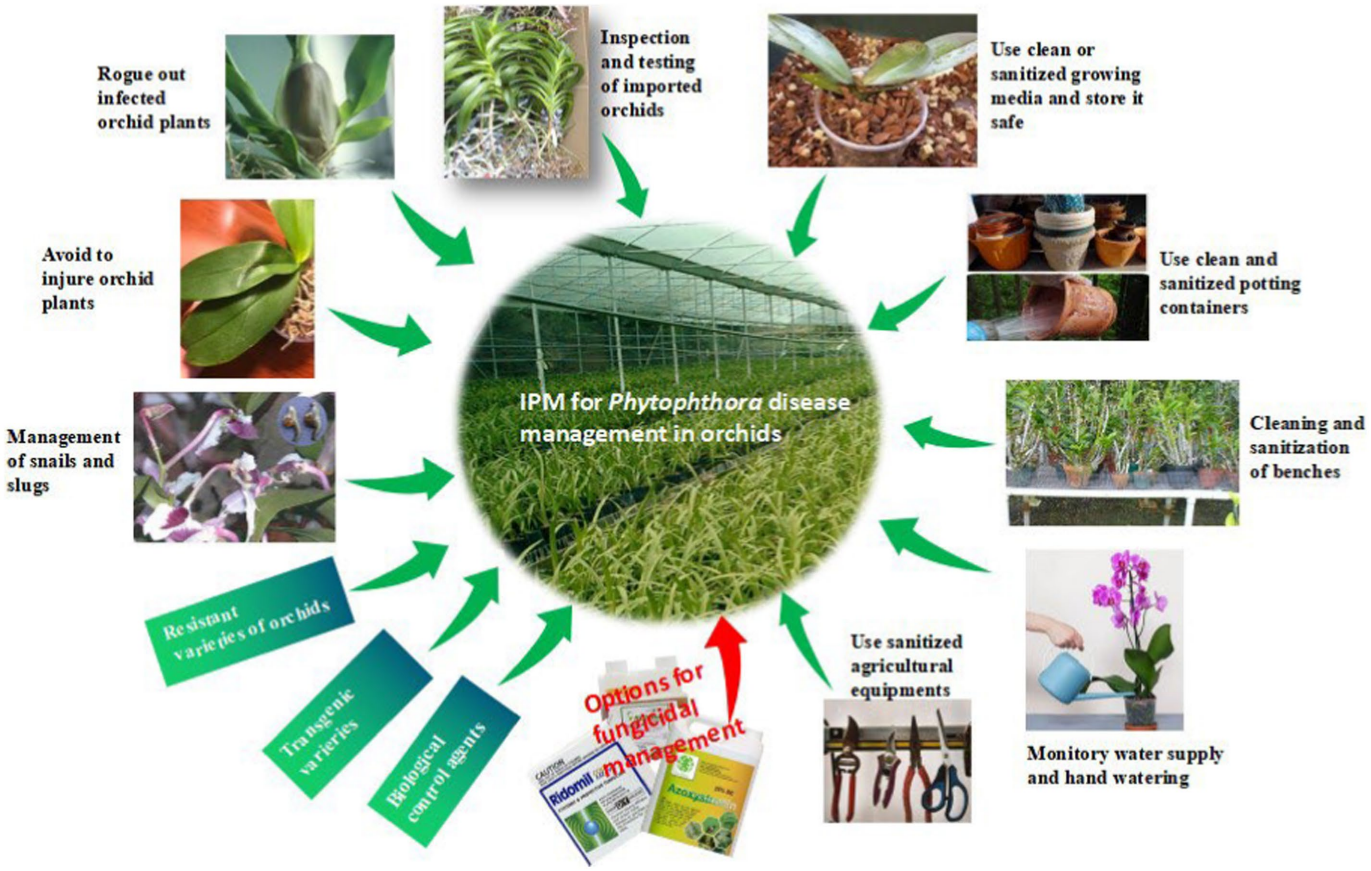
IDM strategies for Phytophthora disease in orchid.

Often, we come across the popular statement “maintenance of beauty is a costly affair.” This statement is aptly applicable to orchids. Unlike openly cultivated fields and other horticultural crops, the orchid crop requires special attention in every stage of the production process, starting from the vegetative phase to flowering and the ultimate sale of the flower to consumers, which certainly involves some extra cost. It may be noted that an exquisitely beautiful orchid hybrid requires special attention for hardening and early establishment in a community pot with proper watering and nutrition after the micropropagation; otherwise, seedlings will be aggravated with so-called water mold, *Phytophthora* or *Pythium*, causing seedling blight, and there may be a chance of losing the costly orchid hybrid developed by the breeder for the first time. Sterilized potting media with optimum watering is required to protect seedlings from damping off during the initial establishment of the seedlings. The integrated management strategies of *Phytophthora* in Orchid are presented in Figure 12, and each of the components is described thereafter.

### Maintenance of optimum water level

*Phytophthora* spp. are water molds; thus, they require water for the development of zoosporangia and zoospores and the further germination of zoospores on the orchid hosts. As a result, water used for irrigation acts as a dispersal medium for the spread of zoospores infecting other plants due to water splashing or watering (Uchida, 1994). Furthermore, zoospores, which are motile in free moisture, are the ultimate infective propagules on orchid hosts and can move fast when free water is readily available. Naturally, high moisture accelerates disease as well as fungal life cycles. The virtual effect of growth and development of pathogens largely aids the development of *Phytophthora* diseases rather than the direct effect of high levels of water on the plant. It is extremely crucial to regulate excess watering and reduce high relative humidity (RH) to manage *Phytophthora* diseases of orchids in small amateur households as well as commercial orchid establishments. All these can be successfully achieved by constructing solid-covered greenhouses or glass houses with good ventilation, reducing prolonged periods of wetness, and using well-drained potting media (Uchida and Aragaki, 1991; Cating et al., 2009). However, growers always need to remain alert with long-term holistic strategies and actions, including preventive, mechanical, and chemical treatment throughout the crop life, for the successful management of *Phytophthora* diseases of orchids. In addition, to keep *Phytophthora* diseases under control, growers should also keep in mind the following points during the production process:

### Growing media and its storage

The growing medium for orchid production plays an important role in the management of disease, as this pathogen is soil-borne. The media used should be fresh and free from the inoculum of *Phytophthora*. Even at the stage of repotting, one should take care of media, as the reuse of old media may potentially contaminate and cause the mortality of seedlings. Freshly purchased growing media should be stored in unopened bags in a closed room. The sealed bags of growing media can also be stored on a concrete floor covered with a polythene sheet. Concrete floors should be periodically washed with bleach (1:3 ratio of sodium hypochlorite to water). Growing media in opened bags, left in an unattended condition for a long time, may get contaminated with species of *Phytophthora*, which may cause the initiation of black rot in the next season if potted with negligence.

### Potting containers

The containers used for the growing of orchids also play an equally important role as media, as they can also carry the potential inoculum of *Phytophthora*. It is advisable to go for new containers wherever it is possible, or the old containers should be treated with a bleaching solution (1:3 ratio of sodium chlorite and water) with agitation for at least 10 min.

### Cleaning and sanitization of benches

Unlike openly cultivated fields and horticultural crops, orchids are not grown on ground soil. They are grown in earthen pots or plastic containers that are placed on raised benches for sufficient aeration. Benches may be made of iron, wooden planks, or a combination of both items. The bench surface should be at least 1 m above the ground soil to avoid splashing from the ground below. It will be better if the benches are kept on the concrete floor to avoid the soil-inhabiting *Phytophthora* from reaching the orchid plants kept on the benches. Single-layer benches are ideal; however, multi-layer horizontal benches are not recommendable because, while watering, excess water might drip on the orchid plants kept on the lower benches from the plants kept on the upper benches. Dripping water will carry the inoculum of *Phytophthora* to lower plants. Sanitization of benches by using available bleaches or disinfectants is also crucial. Several preparations, such as 25% chlorine bleach, 25% pine oil cleaner, 50% rubbing alcohol, 50% denatured ethanol, and 5% quaternary ammonium salts (Cating et al., 2009), are available for sanitization. The wooden portion of benches or wooden benches is difficult to sanitize because they are porous. Regular removal of algae, scum, mildew, and dirt from benches by scrubbing is also required to keep the orchid house clean.

### Quality of water used

Any type of surface water, such as ponds, reservoirs, or even fountain water in hilly areas, can be a potential source of *Phytophthora* zoospores and should not be used unless it is disinfected. In case of hand watering, the hose and wands should be sanitized with a solution of bleach and kept hanging.

### Sanitization of agricultural equipment

Tools such as separating knives, root trimmers (secateurs), and pruning scissors should be sanitized regularly with a bleached solution. Glass, plastic, cloth, or other non-metallic tools, pots, and equipment can be disinfested with freshly prepared 10% Clorox, Physan, or Consan (Uchida and Aragaki, 1991). During repotting, disinfected tools and latex hand gloves may be used. Other potential carriers of *Phytophthora* spores, such as plant transport trailers or carts, should also be sanitized with a bleached solution.

### Season of import of planting materials

As *Phytophthora* spp. are water-loving molds and reproduce zoosporangia and zoospores in high temperatures and saturated moisture conditions, the time of import of orchid materials may also be taken into consideration while importing important breeding lines or extraordinary hybrids for further growth. A study by McMillan et al. (2010) noted that imports of prefinished *Cattleya* orchid liners from Thailand to Florida (USA) during the monsoon season are often infected with *P. cactorum*, whereas *Cattleya* liners imported during the dry season are found to be free from *P. cactorum*.

### Avoidance of wounding orchid plants

Wounds are a prerequisite for the infection of orchid plants by *P. palmivora* (Hine, 1962; Ann, 1995). One should take a lot of care so that the least amount of injuries are inflicted on any part of the orchid plant while they are in transit for sale or exhibition. Furthermore, care should be taken to minimize injuries during various intercultural operations in the greenhouse.

### Isolation of newly introduced orchid plants

New orchid plants introduced in nurseries, greenhouses, or commercial growing hubs may be the potential carriers of *Phytophthora* spp., either on the surface of the plant as a latent infection or carried with the potting media. Therefore, newly introduced orchid plants should be kept in isolation for at least 6 weeks (Cating et al., 2009) to observe the presence of the disease through the manifestation of disease symptoms. The infected orchid plants are removed, and healthy plants are allowed to grow after fungicide application in the growing hub. This reduces the possibility of *Phytophthora’s* introduction into the growing area. Sometimes, introduced or imported orchids with visual symptoms of *Phytophthora* are drenched with fungicides such as Banrot, Truban, Physan 27, Heritage, Stature, Aliette, Subdue Maxx, and Insignia to salvage some of the plants, and severely infected plants are discarded (McMillan et al., 2010).

### Management of snails and slugs in the orchidarium

Snails and slugs invade the orchid hubs and feed on the green parts of the orchid plants. They not only damage the orchid crops by feeding on leaves and flowers but also work as passive carriers of *Phytophthora* when they enter the commercial growing hub of orchids. *Helix* spp. (snails) and *Philomycus* spp. (slugs) are considered potential agents for the spread of *Phytophthora* pathogens in Taiwan (Hsieh, 1984). These soft-bodied animals may carry pathogen zoospores either on their bodies or by ingesting zoospores during the feeding of diseased plant tissue and later excreting viable zoospores on the plant surface (Uchida and Aragaki, 1991).

### Chemical disease management

Regular practice of the above-mentioned preventive and mechanical control measures can minimize the intensity of *Phytophthora* disease in orchids in commercial growing hubs but may not always control a cent percent of the disease. In this condition, options for the application of chemical fungicides can be considered. Early-diagnosed disease can be treated by drenching the plant with a protective fungicide such as Turban or Terrazole. For advanced stages of the disease, systemic fungicides such as Aliette or Subdue will be more effective. Captan, Dithane M 45, and Physan 20 are also recommended for the control of black rot (Jones, 2002). *Cattleya* leaf and flower bud infection caused by *P. cactorum* was effectively managed with the preventive sprays of Aliette, Insignia, Stature, and Subdue Maxx. However, preventive drenching with fungicides Aliette, Banrot, Heritage, Insignia, Shield Brite, Stature, Subdue Maxx, and Turban is found to be significantly effective for controlling *P. cactorum* in community pots for *Cattleya* seedlings (McMillan et al., 2009). Various fungicides used for the management of *Phytophthora* diseases of orchids in various countries are listed below (Table 10).

**Table 10:** Efficacy of different fungicides on *Phytophthora* diseases of orchids.

Sl. no.	Chemical name	Trade name	Type of fungicide	Mode of application	Response	Effective against	Phytotoxicity to orchids	Reference
1.	Fosetyl-aluminum potassium	Aliette, Flanker	Systemic	Spray	+++	Pc, pp	Nil	Lim and Nio (1983); McMillan (1983); McMillan et al. (2009); Kawate and Sewake (2010)
2.	Pyraclostrobin	Insignia, pageant, empress intrinsic	Systemic	Spray, drenching	+++	pc	NR	McMillan et al. (2009); Kawate and Sewake (2010)
3.	Thiabendazole	Shield Brite	Systemic	Drenching	+++	pc	NR	McMillan et al. (2009)
4.	Dimethomorph	Stature DM	Systemic	Spray, drenching	+++	pc	NR	McMillan et al. (2009)
5.	Metalaxyl	Subdue MAXX, Ridomil	Systemic	Seedling dips, spray, drenching	+++	Pc, pp	Nil	Lim and Nio (1983); McMillan (1983); McMillan et al. (2009)
6.	Metalaxyl plus mancozeb	Ridomil MZ	Systemic + contact mixture	Spray	+++	pp	nil	Lim and Nio, (1983)
7.	Metalaxyl+ Mancozeb	Metalaxyl MZ (G)	Systemic	Soil application	Excellent	pp	NR	Leu (1994)
8.	Etridiazole + thiophanate methyl	Banrot	Contact + systemic	Drenching	+++	pc	NR	McMillan et al. (2009)
9.	Azoxystrobin	Heritage,	Systemic	Drenching	+++	pc	NR	McMillan et al. (2009)
10.	Etridiazole	Truban, Terrazole	contact	Drenching	+++	pc	NR	McMillan et al. (2009); Kawate and Sewake (2010)
11.	8-hydroxyquinoline sulphate	Bioquin700	Systemic	Seedling dips	+++	pc	Nil	McMillan (1983)
12.	Sodium o-hydroxy diphenyl	Natriphene	Systemic	Seedling dips	+++	pc	nil	McMillan (1983)
13.	Difolatan	Captafol	Systemic	Spray	+++	pp	Nil	Lim and Nio (1983)
14.	Mefenoxam (Metalaxyl-M)	Subdue, Metastar, Ariel	Systemic		++	*Phytophthora*	NR	Kawate and Sewake (2010)
15.	Fludioxonil + mefenoxam	Hurricane	Contact + systemic	Drench	++	*Phytophthora*	NR	Kawate and Sewake (2010)

+++ Significantly effective. Pc = Phytophthora cactorum, Pp = Phytophthora palmivora, NR nor reported.

## Future perspectives

Since the aegis of wild orchid hunting, much has been achieved, from the domestication of wild orchids to their commercialization in global markets. Once upon a time, beautiful orchids were within the grasp of only the elite classes of people, but now it is brought to the open market for all classes due to the concerted effort of orchid biologists and orchid fraternities. At the same time, orchid health management, with special reference to *Phytophthora* diseases, has also progressed substantially. However, in the present context of soil and water pollution and global health hazards, eco-friendly options for disease management, *viz.*, the development of resistant hybrids/cultivars, biological disease management, transgenic approaches, and RNAi technology, need encouragement.

### Resistance sources of orchids against *Phytophthora* diseases

In the 21st century, scientists from various sections, such as agriculture or horticulture, are in search of resistant sources either in the existing landraces or exotic world germplasm for prospective use in the development of durable resistant hybrids (maybe they are orchids or other crops). An extensive survey of global literature indicates that at present there are no resistant varieties of any category of orchids against *Phytophthora*. Even so, there is no report of natural resistance in wild species or landraces of orchids against *Phytophthora* diseases. Attempts should be made for a systemic search for any novel resistant gene among wild and landraces of orchids that can be incorporated into popular susceptible hybrids/cultivars using suitable molecular techniques. Alternatively, tolerance of orchids could be enhanced against *Phytophthora* spp. in question by the insertion of chitinase and glucanase genes. Since orchids are mostly propagated using tissue culture techniques, an attempt was made for the *in vitro* selection of *Dendrobium* “Earsukul” PLBs (protocorm-like bodies) resistant to *P. palmivora* using *P. palmivora* CF (culture filtrate) containing α-elicitin. Several putative mutants resistant to *P. palmivora* in detached leaf assay have been selected in Thailand. One such *Dendrobium* mutant resistant to all isolates of *P. palmivora* is “SUT13E18-A,” which can be used as a resistant source in a future breeding program (Khairum et al., 2016, 2018).

### Biological control of *Phytophthora* diseases of orchids

Although a large number of country-specific commercial formulations of biocontrol agents are available for the management of plant diseases (Sevugapperumal et al., 2016; Hyder et al., 2017). However, very limited bioagents are available for the management of *Phytophthora* diseases in orchids worldwide. In a recent study, *Pseudomonas aeruginosa* RS1 was reported to have a good inhibitory effect on *P. palmivora*, causing orchid black rot. The antifungal proteins from *Pseudomonas aeruginosa* RS1 were identified as an active compound for inhibiting the growth of *P. palmivora*. The effective protein molecules are identified through LC/MS analysis. The proteins are found to be closely identified with three broad groups, such as catalase, chitin-binding protein, and protease. Partially purified proteins from *P. aeruginosa* RS1 caused abnormal growth and hyphal elongation in *P. palmivora* (Sowanpreecha and Rerngsamran, 2018). Similarly, in a parallel study, *Streptomyces similanensis* strain 9X166 has shown high antagonistic activity against *P. palmivora* which causes black rot in orchids in both *in vitro* and *in vivo* assays. Endo β-1,3-glucanase produced by the actinomycetes *Streptomyces similanensis* is the active principle for antagonistic activity. Consequently, *Streptomyces* sp. 9X166 culture filtrate, containing β-1,3-glucanase, can degrade freeze-dried as well as the living mycelium of *P. palmivora*. Therefore, β-1,3-glucanase-producing *Streptomyces* sp. can be an effective biocontrol agent for black rot of orchids (Sakdapetsiri et al., 2016). For mass production of *Streptomyces similanensis* 9X166, solid-state fermentation using agro-industrial substrates is being standardized in Thailand. Up to 60 days, the product retained 10^6^CFU/g of *Streptomyces similanensis* 9X166 in a dried solid which can effectively inhibit cent percent of *P. palmivora* in living orchids (Sakdapetsiri et al., 2019).

### Biotechnological and transgenic approaches for the *Phytophthora* management in orchids

Needless to say, biotechnological interventions have immensely contributed to the improvement of orchids in various aspects: phylogenetic studies, embryology, tissue culture, micropropagation, somaclonal variation, germplasm conservation, and mycorrhizal technology in orchid seed germination and natural establishment and diagnosis of orchid viruses (Hossain et al., 2013). Genetic engineering has further contributed to the improvement of complex orchid traits such as flower color, vase life, and genetic control of flower morphogenesis through the transformation of orchids (Hossain et al., 2013), where conventional breeding could not satisfactorily enlighten the stable path. However, biotechnological interventions, including transgenic approaches, did not contribute much to the development of *Phytophthora* disease-resistant varieties in orchids, except in a few cases of viral and bacterial diseases. Recently, Sjahril et al. (2006) carried out an *Agrobacterium*-mediated transformation in *Phalaenopsis* orchid (*Phalaenopsis* Wataboushi ‘#6.13’) with the incorporation of the wasabi defensin gene (WjAMP-1) in Japan. The transformed *Phalaenopsis* orchid overexpressed resistance to soft rot pathogen *Erwinia carotovora* subsp. *carotovora.* In Indonesia, a different method, the particle bombardment approach, was successfully used to incorporate the wasabi defensin gene into *Phalaenopsis* orchids for the development of transgenic *Phalaenopsis amabilis* resistant to soft rot bacterial *Erwinia carotovora* subsp. *carotovora* (Mariani, 2016). A transgenic *Oncidium* orchid resistant to soft rot bacteria (*Erwinia carotovora* subsp. *carotovora*) has been developed by incorporation of sweet pepper ferredoxin-like protein (*pflp*) gene into *Oncidium* (cv. Sherry Baby OM 8) using *Agrobacterium tumefaciens* as a vector in China (Liau et al., 2003). Transgenic *Dendrobium* orchids cloned with the CymMV coat protein gene expressed *Cymbidium* virus-resistant capacity (Petchthai et al., 2015). All of these can, therefore, strongly support the idea that the genetic transformation of orchids was very successful in the development of durable resistance against individual phytopathogens. At the same time, there is also a graceful breakthrough by a different group of researchers to develop transgenic orchids with multiple disease resistance against more than one phytopathogen by gene staking through double transformation. In Taiwan, transgenic *Phalaenopsis* orchids, which were developed by the incorporation of CymMV coat protein (CP) and sweet pepper ferrodoxin-like protein (*pflp*) by double transformation, expressed dual resistance to the Cymbidium mosaic virus and the soft rot bacterium *Erwinia carotovora* subsp. *carotovora* (Chan et al., 2005). However, a breakthrough is certainly zero when one narrows down its search to the contribution of biotechnology, including transgenic approaches for the development of disease-resistant orchids against any species of *Phytophthora*. To date, there is not a single hybrid or transgenic orchid in the global market to showcase that it is resistant to *Phytophthora* diseases of orchids. Here, the focus needs to be on the future of supporting the orchid business *vis a vis* world orchid trade.

## Concluding remarks

Since the potato late blight outbreak caused by *Phytophthora infestans* in 1845 and the worst Irish Potato Famine (1845–1849) in Europe in the 19th century, intensive studies have been done on host–pathogen interaction, resistant breeding, identification of races/pathotypes, identification of R-genes, and the development of genetically modified crop varieties involving *Phytophthora* with many of its field and horticultural crops worldwide. However, there is a lack of information regarding host–pathogen interaction between any *Phytophthora* sp. and orchid host in the entire Plant Pathology literature. About a century has passed since the first report of *Phytophthora palmivora* infection on *Dendrobium maccarthiae* in Ceylon in 1921 (Petch, 1921), and hardly any scientific literature is there to support the status of race or pathotype of any *Phytophthora* species infecting orchids. For developing resistant varieties of orchids against *Phytophthora* spp. in any country, defining the prevalent races or pathotype structures is a prerequisite. A lot of literature is available on conventional breeding for the improvement of flower characteristics, such as big, durable, and fascinating flowers, in addition to the enhancement of quality features such as flower color, vase durability attribute, shape, and architecture and genetic engineering also showed a successful breakthrough for changing complex characters in orchids, such as novel flower color (Chia et al., 2001) and increased vase life (Chia et al., 2001), with the identification of specific genes involved for those characters. Although more than one hundred thousand interspecific, intergeneric, or multi-generic hybrids of orchids were developed through conventional breeding worldwide, breeding for the development of resistant varieties against *Phytophthora* diseases of orchids is still absent in the orchid industry. Breakthroughs on the patterns of inheritance of resistance against *Phytophthora* spp. in orchids are yet to come. Data on the availability of the R-gene in orchids also remained obscure. Identification of resistance sources in orchid species and landraces is needed to be strengthened; otherwise, the development of durable resistant orchid hybrids against any *Phytophthora* sp. will remain a dream.

## Author contributions

TB conceived and designed the study and wrote the first draft of the manuscript with a graphical presentation and tables. PD supervised the final draft of the manuscript, designed the graphical abstract, and arranged the table. RK prepared the phylogenetic trees. MH, MM, AC, GD, MK, and RW checked the drafted manuscript. All authors contributed to the article and approved the submitted version.
