# Supplementary figures and images for: Destructive Phytophthora on orchids: current knowledge and future perspectives
Source: Front Microbiol. 2024 Jan 5;14:1139811. doi: 10.3389/fmicb.2023.1139811 (PMC10810131; doi:10.3389/fmicb.2023.1139811)

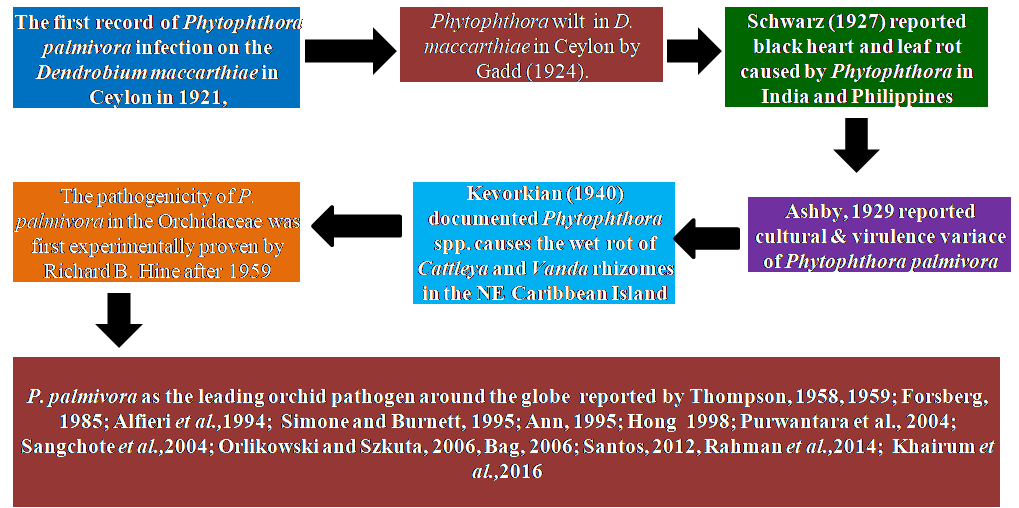

Supplement: Supplementary file 1 [file Image_1.PNG]

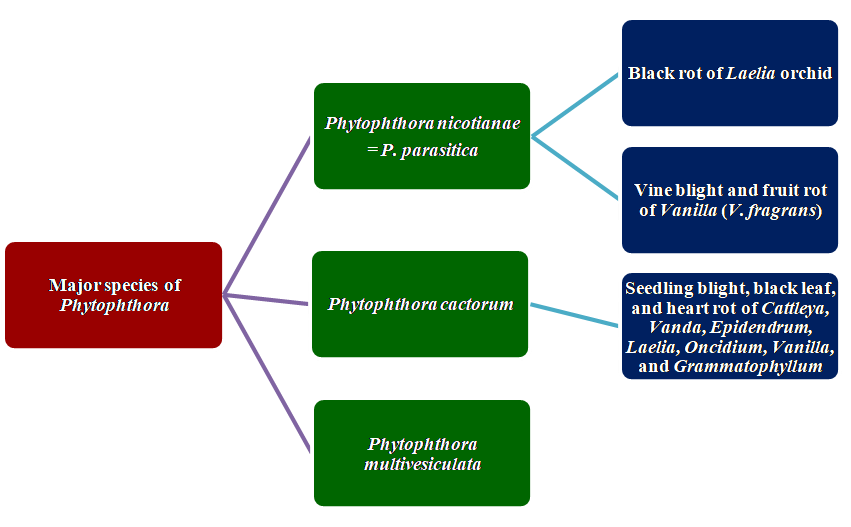

Supplement: Supplementary file 2 [file Image_2.PNG]
