# Supplementary material for: Destructive Phytophthora on orchids: current knowledge and future perspectives
Source: Front Microbiol. 2024 Jan 5;14:1139811. doi: 10.3389/fmicb.2023.1139811 (PMC10810131; doi:10.3389/fmicb.2023.1139811)

**Taxonomy of *Phytophthora***

| **Domain- Eukarya** | | | | | | | |
| --- | --- | --- | --- | --- | --- | --- | --- |
|  | **Kingdom- Chromista** | | | | | | |
|  |  | **Phylum – Oomycota** | | | | | |
|  |  |  | **Class- Oomycetes** | | | | |
|  |  |  |  | **Order -Peronosporales** | | | |
|  |  |  |  |  | **Family- Peronosporacae** | | |
|  |  |  |  |  |  | **Genus-*Phytophthora*** | |
|  |  |  |  |  |  |  | **Species-*palmivora*** |

**Taxonomy of *Phytophthora***


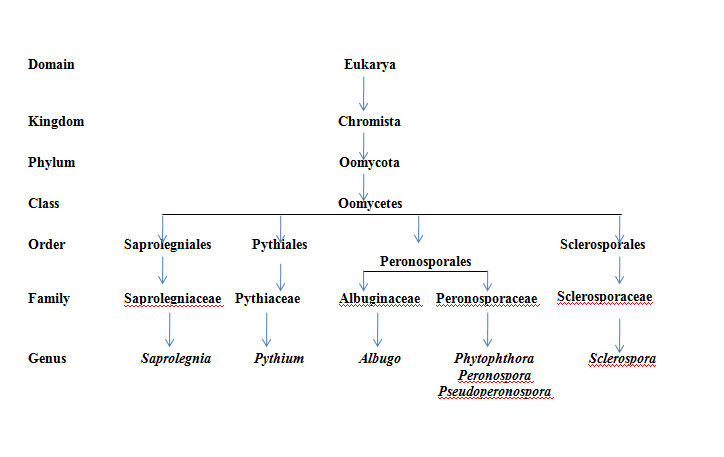

Supplement: Supplementary file 3 [file Table_1.DOCX]
